# Interplay between Porphyromonas gingivalis Hemophore-Like Protein HmuY and Kgp/RgpA Gingipains Plays a Superior Role in Heme Supply

**DOI:** 10.1128/spectrum.04593-22

**Published:** 2023-02-08

**Authors:** Michał Śmiga, Paulina Ślęzak, Mateusz Wagner, Teresa Olczak

**Affiliations:** a Laboratory of Medical Biology, Faculty of Biotechnology, University of Wrocław, Wrocław, Poland; Griffith University

**Keywords:** *Porphyromonas gingivalis*, HmuY, HusA, gingipain, hemophore-like protein, iron homeostasis, heme homeostasis

## Abstract

To acquire heme as a source of iron and protoporphyrin IX, Porphyromonas gingivalis uses gingipains, Hmu, and Hus systems. The aim of this study was to assess the correlation between the production and function of the most important virulence factors of P. gingivalis involved in heme supply, namely, hemophore-like proteins (HmuY and HusA) and gingipains. Respective mutant strains were used, and the expression of genes at the transcript and protein levels, as well as the importance of these genes’ products for virulence potential, was examined. We found that HmuY and Kgp/RgpA gingipains are among the main P. gingivalis virulence factors synergistically engaged in heme supply. Their expression is related mainly when P. gingivalis grows in conditions rich in iron and heme sources, resembling those found in severe periodontitis. We confirmed that HmuY production is strictly dependent on the availability of heme and iron in the external environment, whereas we did not observe such dependence in the production of HusA. Moreover, we found that the HmuY protein can easily sequester heme from the HusA protein. The only correlation in the production of HmuY and HusA hemophore-like proteins could occur in P. gingivalis grown in conditions rich in iron and heme sources, mimicking an environment typical for severe periodontitis. Based on our observations, we suggest that HmuY is the major heme-binding protein produced by P. gingivalis, especially in iron- and heme-depleted conditions, typical for healthy periodontium and the initial stages of infection. The HusA protein could play a supporting role in P. gingivalis heme uptake.

**IMPORTANCE** Altered or disturbed mutualism between oral microbiome members results in dysbiosis with local injuries and subsequently in systemic diseases. Periodontitis belongs to a group of multifactorial infectious diseases, characterized by inflammation and destruction of tooth-supporting tissues. Porphyromonas gingivalis is considered the main etiologic agent and keystone pathogen responsible for developing advanced periodontitis. As part of the infective process, P. gingivalis must acquire heme to survive and multiply at the infection site. Analysis of the mutual relationship between its main virulence factors showed that heme acquisition in P. gingivalis is a complex process in which mainly the Hmu system, with the leading role played by the HmuY hemophore-like protein, and Kgp and RgpA gingipains prefer cooperative interplay. It seems that the Hus system, including HusA hemophore-like protein, could be involved in another, so far uncharacterized, stage of iron and heme supply.

## INTRODUCTION

The healthy oral cavity is inhabited by well-balanced microbiome members, with species of Streptococcus being the most abundant (1, 2). Analysis of bacterial species associated with periodontal diseases has revealed the increased abundance of those belonging to the *Bacteroidetes* phylum, with a prevalence of “red complex” members (Porphyromonas gingivalis, Tannerella forsythia, and Treponema denticola), which are associated with the clinical features of periodontal diseases (3–6). Among them, P. gingivalis is considered the main etiologic agent and keystone pathogen responsible for disruption of host homeostasis, increasing polymicrobial biofilm formation, causing dysbiosis, and leading to the development of periodontitis (7–9). Recent studies demonstrated that periodontal anaerobes, mainly P. gingivalis, also play an important contributory role in systemic diseases, such as diabetes, rheumatoid arthritis, atherosclerosis, cardiovascular and respiratory diseases, and Alzheimer’s disease (10–18).

In its normal habitats (the periodontal pocket and gingival sulcus), P. gingivalis is a constituent of a multispecies biofilm, where it experiences fluctuations in the availability of nutrients, including iron and heme, both required for the establishment and progression of infection. P. gingivalis neither produces and utilizes siderophores to gain iron nor produces secreted hemophores to gain heme, and it is absolutely dependent on heme due to the lack of a functional heme biosynthesis pathway (19). However, *in vivo* free heme (a major iron and protoporphyrin IX source) is not readily available because it is toxic and therefore is rapidly bound by host heme-scavenging proteins (20–23). To overcome these significant barriers to heme acquisition, microorganisms have evolved sophisticated heme acquisition systems (24).

P. gingivalis uses a complex heme uptake mechanism that enables this bacterium to adapt to the changing environmental conditions that vary in the source and amount of available heme and iron (19). The best-characterized heme uptake system is that encoded by the *hmu* operon (Hmu system), allowing production of HmuY, a heme-binding hemophore-like protein, HmuR, a typical TonB-dependent receptor transporting heme through the outer membrane, and four proteins with unknown function (19). HmuY is the first representative of a novel family of proteins with unique structural and heme-binding properties (25–27). P. gingivalis produces higher levels of HmuY when the bacterium grows in low-iron and -heme culture media, as a biofilm constituent, and intracellularly in host cells (26, 28–31). The HmuY protein is associated with both the bacterial outer membrane and outer membrane vesicles (OMVs) through a lipid anchor (26, 30, 32–34). In order to efficiently bind and deliver heme to the bacterial cell, HmuY is released from the lipid anchor by specific cleavage performed through the limited proteolytic processing by a lysine-specific cysteine protease (Kgp gingipain) (26, 30). Our work has shown that HmuY protein is resistant to several proteases, including P. gingivalis gingipains (26, 35), as well as host proteases, including neutrophil elastase (12), which allows its distribution and accumulation in the host environment. Importantly, HmuY is expressed *in vivo* in patients with chronic periodontitis (5, 6). We also demonstrated that significantly increased levels of anti-HmuY antibodies exist in sera of patients with periodontitis (36).

The second well-characterized P. gingivalis heme acquisition system is that encoded by the *hus* operon (Hus system) (37, 38). HusA is a hemophore-like protein, and HusB is a typical TonB-dependent outer membrane receptor, whereas the functions of HusC and HusD are unknown. The Hus protein is produced in the form attached to the outer membrane of the bacterial cell and OMVs, as well as in a soluble extracellular form (38). In contrast to HmuY (25, 26), HusA prefers binding metal-free porphyrins without heme iron coordination (38).

The third main component involved in P. gingivalis heme acquisition comprises gingipains, cell surface-anchored or soluble cysteine proteases, responsible for up to 85% of the total extracellular proteolytic activity of this bacterium (19, 39–41). This activity is required for nutrient acquisition for this asaccharolytic pathogen and for avoiding the host immune response through cleavage of cell surface receptors, signaling via protease-activated receptors, and inactivation of cytokines and components of the complement system (42–46). Arginine-specific gingipains (RgpA and RgpB) are encoded by the *rgpA* and *rgpB* genes, whereas Kgp is encoded by the single *kgp* gene (42, 47). The predominant form of Kgp and RgpA is a complex of a catalytic domain with hemagglutinin/adhesion domains. Their catalytic regions are divergent, but hemagglutinin domains are similar to each other (42, 47, 48). Some regions of the hemagglutinin domains are also encoded by the *hagA* gene (48, 49). Soluble Kgp and RgpA bind hemoglobin with higher efficiency than heme, other metalloporphyrins, and porphyrins (50, 51). Binding to metalloporphyrins and porphyrins is similar, suggesting that interaction between these compounds and gingipains does not involve iron. Moreover, Kgp can more effectively degrade host hemoproteins than RgpA and RgpB (52, 53). Further studies demonstrated that Kgp is not absolutely required for heme acquisition from hemoglobin but makes this process more efficient (35, 54).

Our previous studies revealed that P. gingivalis displays a novel heme acquisition mechanism whereby oxyhemoglobin is first oxidized to methemoglobin, facilitating heme release. To generate methemoglobin, P. gingivalis involves mostly RgpA (35). The bacterium is then able to fully proteolyze the more susceptible methemoglobin substrate to release free heme, mostly through P. gingivalis Kgp activity, and via direct heme sequestration by HmuY. *In vivo*, HmuY expression was correlated with higher expression of other important P. gingivalis virulence factors, including gingipains (5, 6, 12), suggesting that using the synergistic action of the HmuY protein and gingipains is a very important part of the strategy of P. gingivalis to obtain heme.

The aim of this study was to broaden our knowledge of the correlation between production and function of the most important virulence factors of P. gingivalis engaged in heme supply, namely, hemophore-like proteins (HmuY and HusA) and gingipains. For this purpose, respective mutant strains were used, and the expression of genes at the transcript and protein levels, as well as the importance of these genes’ products for virulence potential, was examined. Our data demonstrated that HmuY and Kgp/RgpA gingipains count among the main P. gingivalis virulence factors synergistically engaged in heme supply, whereas the involvement of HusA in this process requires further clarification.

## RESULTS

### Influence of the lack of components of the main heme supply system on P. gingivalis growth phenotype and virulence.

**FIG 1:**
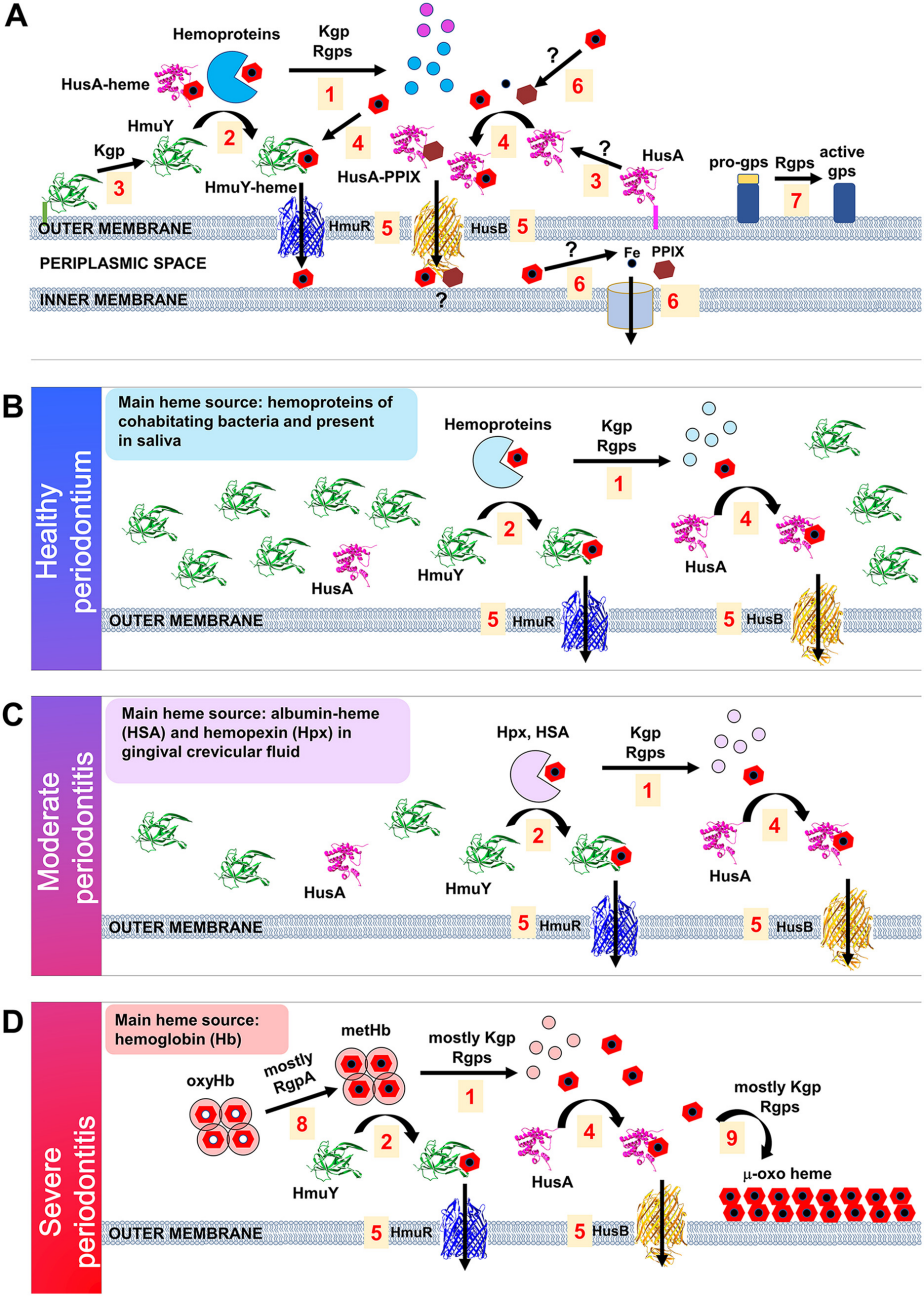
Schematic presentation of the interplay between the main components of the heme acquisition system of P. gingivalis used in different stages of infection. (A) Best-characterized components of the main P. gingivalis heme acquisition system. Host hemoproteins and heme-binding proteins produced by cohabitating bacteria serve as a main source of heme for P. gingivalis. Heme can be released from hemoproteins by proteolysis performed by gingipains (gps) (A, 1) or directly sequestered from hemoproteins, mainly by HmuY hemophore-like protein (A, 2). To facilitate heme binding, Kgp-limited proteolysis releases HmuY from the lipid anchor (A, 3), and soluble HmuY protein could be spread and more efficient in heme binding. A similar process occurs in the case of HusA protein, but the protease releasing HusA protein is not known (A, 3). Released heme can be bound directly by HmuY and HusA (A, 4) and then transferred to TonB-dependent outer membrane receptor from HmuY to HmuR and from HusA to HusB (A, 5). Further process of heme transport and iron release is not known (A, 6) but might involve HusA, which preferentially binds iron-free protoporphyrin IX (PPIX) (A, 4). Gingipains also facilitate heme uptake through other processes, such as engagement in their own maturation (A, 7). (B) In healthy periodontium, access to heme is limited, and the HmuY protein is produced at high levels. Heme bound to hemoproteins present in saliva and hemoproteins produced by cohabitating bacteria is acquired by P. gingivalis through the proteolytic activity of gingipains (B, 1) and heme sequestration by HmuY (B, 2). (C) In moderate periodontitis, an additional source of heme comes from increased flow of gingival crevicular fluid containing serum hemoproteins (hemopexin [Hpx] and albumin in complex with heme [HSA]), and HmuY is produced at moderate levels. Heme bound to hemoproteins present in saliva, and hemoproteins produced by cohabitating bacteria are acquired by P. gingivalis through the proteolytic activity of gingipains (C, 1) and heme sequestration by HmuY (C, 2). (D) In severe periodontitis, when bleeding occurs, P. gingivalis uses hemolysins, hemagglutinins, and gingipains to obtain heme from hemoglobin (Hb) released from erythrocytes. The HmuY protein is produced at lower levels; however, sequestration of heme by HmuY (D, 2) from oxyhemoglobin (oxyHb) is facilitated by its oxidation to methemogobin (metHb) due to gingipain (mainly RgpA) activity (D, 8). Excess of heme is deposited on the P. gingivalis cell surface in the form of μ-oxo heme (D, 9) produced with the involvement of gingipains (mainly Kgp).

**FIG 2:**
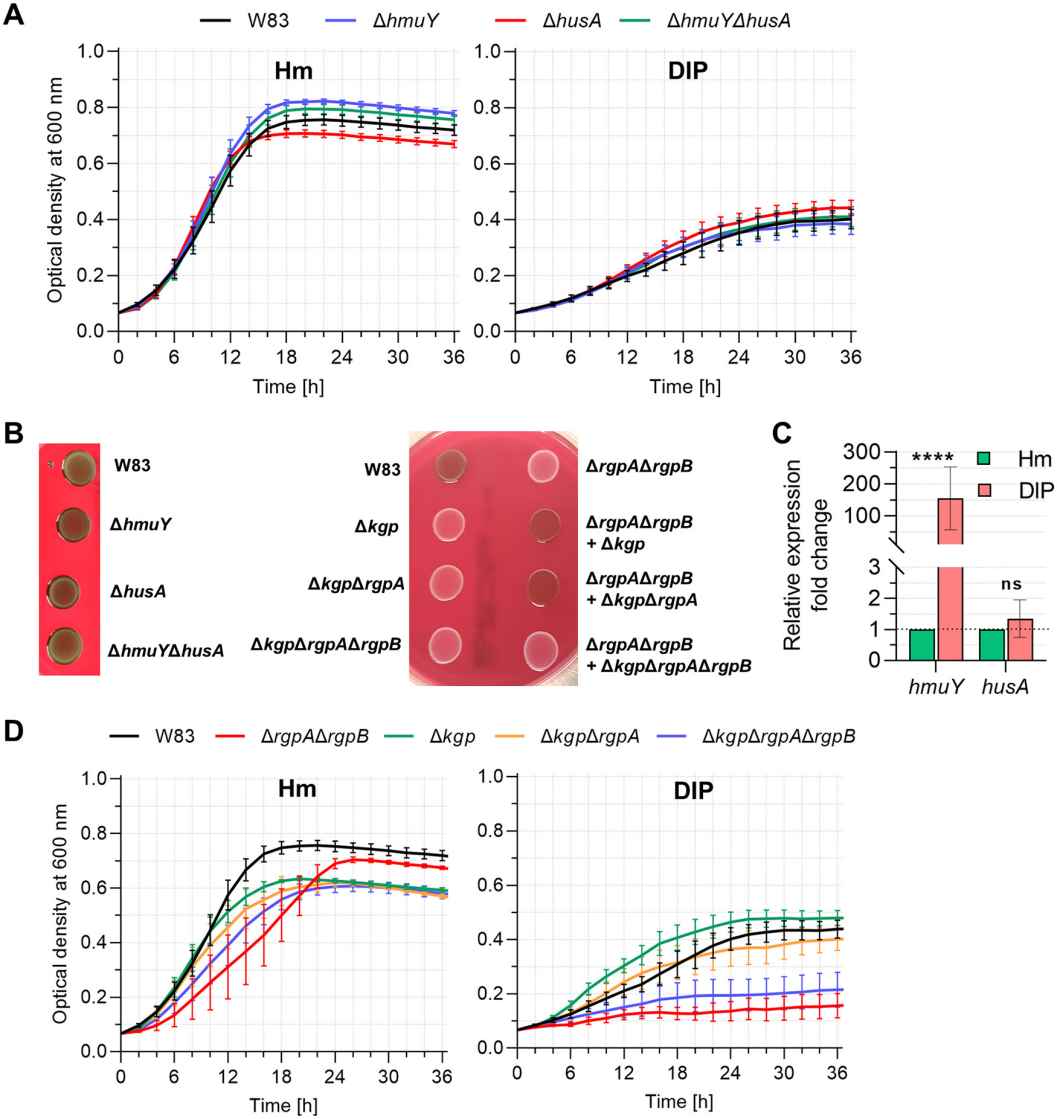
Comparison of growth phenotype of P. gingivalis and *hmuY* or *husA* transcript level. (A and D) Optical density at 600 nm of liquid bacterial cultures of W83 strain and W83-derived mutant strains lacking hemophore-like proteins (A) or gingipains (D) maintained in iron- and heme-rich conditions (Hm) or in iron- and heme-depleted conditions (DIP), monitored for 36 h using Stratus plate reader. Mean values ± standard error (mean ± SE) from three independent measurements are shown. (B) Pigment formation on blood agar plates was observed by visual inspection after 5 days. (C) Relative transcript levels of *hmuY* and *husA* were determined using RT-qPCR in the P. gingivalis W83 wild-type strain grown for 24 h in iron- and heme-depleted conditions (DIP) in comparison to bacteria grown in iron- and heme-rich conditions (Hm); ns, statistically not relevant; ****, *P < *0.0001.

P. gingivalis is a heme auxotroph and requires heme for proliferation and virulence (19). Disturbances in the functioning of components of its main heme acquisition system allow one to determine their role in heme supply (e.g., references 25, 28, 29, and 31). To better define the relationship between hemophore-like proteins, HmuY and HusA, and gingipains at different stages of P. gingivalis infection (Fig. 1), we analyzed deletion mutant strains (Table 1; see Fig. S1 in the supplemental material) cultured in conditions differing in iron and heme availability (Fig. 2A and B). We believe that this approach allowed us to better understand not only the role played by HmuY, HusA, and gingipains in P. gingivalis heme acquisition but, importantly, their mutualism in relation to the virulence potential of this pathogen. Similar to the A7436 strain routinely used in our previous studies, the W83 strain employed in the present study produced significantly higher levels of mRNA encoding HmuY when bacteria had been starved of iron and heme (DIP medium, basal medium without added heme and supplemented with iron chelator, 160 μM dipyridyl) in relation to bacteria cultured in iron- and heme-rich conditions (Hm medium, basal medium supplemented with heme) (Fig. 2C). In contrast, levels of mRNA encoding the second hemophore-like protein, HusA, did not change significantly under such conditions (Fig. 2C).

**TABLE 1:** P. gingivalis strains constructed and/or used in this study

Strain	Deletion or insertion	Rgp catalytic activity	Kgp catalytic activity	Gingipain hemagglutinin activity	Antibiotic cassette(s) (antibiotic concn [μg/mL])	Source or reference
W83	Wild type	+	+	+	None	Laboratory collection
Δ*kgp*	*kgp*^Δ598–1732^Tc^r^	+	−	+	Tetracycline (1)	55
Δr*gpA*Δr*gpB*	*rgpA-rgpB410-BEm* ^r^	−	*+*	*+*	Chloramphenicol (5), erythromycin (5)	56
Δ*kgp*Δ*rgpA*	*kgp*^Δ598–1732^Tc^r^*rgpA*-Cm^r^	+	−	−	Tetracycline (1), chloramphenicol (5)	58
Δ*kgp*Δ*rgpA*Δ*rgpB*	*kgp*^Δ598–1732^Tc^r^*rgpA*-Cm^r^*rgpB*^Δ410–507^Em^r^	−	−	−	Tetracycline (1), erythromycin (5), chloramphenicol (5)	57
Δ*hmuY*	*hmuY*^Δ^Em^r^	*+*	*+*	*+*	Erythromycin (5)	This study
Δ*husA*	*husA*^Δ^Tc^r^	*+*	*+*	*+*	Tetracycline (2)	This study
Δ*hmuY*Δ*husA*	*hmuY*^Δ^Em^r^*husA*^Δ^Tc^r^	*+*	*+*	*+*	Erythromycin (5), tetracycline (2)	This study

To characterize the phenotype of the mutants, we first examined P. gingivalis growth rate. No statistically significant differences were observed in the growth curves between the wild-type W83 strain and the Δ*hmuY*, Δ*husA*, and Δ*hmuY* Δ*husA* strains cultured in liquid Hm and DIP media (Fig. 2A). Additionally, no significant changes in growth and pigmentation were observed when strains were cultured on anaerobic blood agar (ABA) plates (Fig. 2B). Although characterization of the gingipain mutant strains has been reported previously (55–58), confirmation of their phenotype under conditions used in our study was necessary to properly explain our findings. Compared to the wild-type W83 strain, as well as to mutant strains lacking functional *hmuY* and/or *husA* genes, growth of all gingipain mutant strains in Hm medium was delayed (Fig. 2D). When bacteria have been starved of iron and heme (DIP), growth of Δ*rgpA* Δ*rgpB* and Δ*kgp* Δ*rgpA* Δ*rgpB* strains was decreased (Fig. 2D). Compared to the wild-type W83 strain, the pigmentation of all gingipain mutant strains grown on ABA plates was significantly decreased (Fig. 2B).

**FIG 3:**
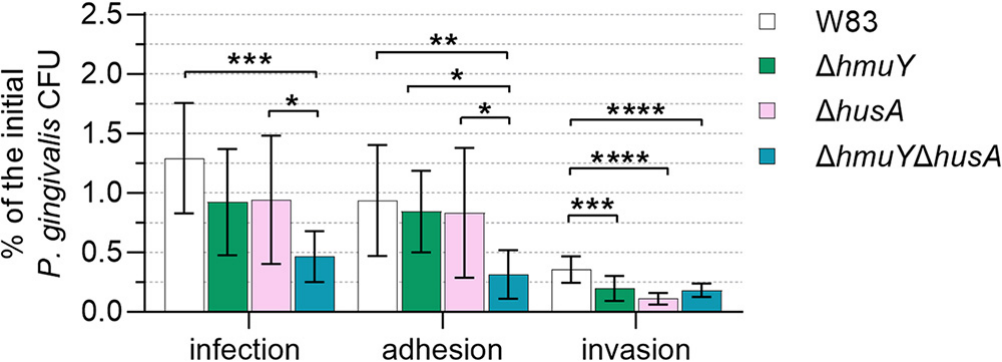
Infection of human host cells by P. gingivalis. The ability to infect, invade, and adhere to gingival keratinocytes of the wild-type W83 and Δ*hmuY*, Δ*husA*, and Δ*hmuY* Δ*husA* mutant strains was analyzed using a P. gingivalis-host cell coculture model. The number of viable bacteria was shown as the percentage of the initial P. gingivalis CFU added to the gingival keratinocytes. Adhesion, live bacteria attached to keratinocytes; invasion, live bacteria that invaded keratinocytes; infection, the total number of live bacteria that invaded and adhered to keratinocytes. The experiment was performed three times in two biological replicates. *, *P < *0.05; **, *P < *0.01; ***, *P < *0.001; ****, *P < *0.0001.

We further aimed to analyze which of the hemophore-like proteins plays a more important role in infection of gingival keratinocytes and whether these proteins may work in cooperation in this process. Our results confirmed that deletion of the *hmuY* gene and/or *husA* gene resulted in general lower infection ability (Fig. 3). Inactivation of the *hmuY* and/or *husA* genes significantly affected the invasion ability of keratinocytes by P. gingivalis. Also, adhesion to keratinocytes was decreased in the case of the Δ*hmuY* Δ*husA* strain compared to W83, Δ*hmuY*, and Δ*husA* strains. Our results demonstrated that both hemophore-like proteins could be important for *in vivo* survival and proliferation of P. gingivalis.

### Influence of HmuY and HusA proteins on other P. gingivalis heme acquisition components.

**FIG 4:**
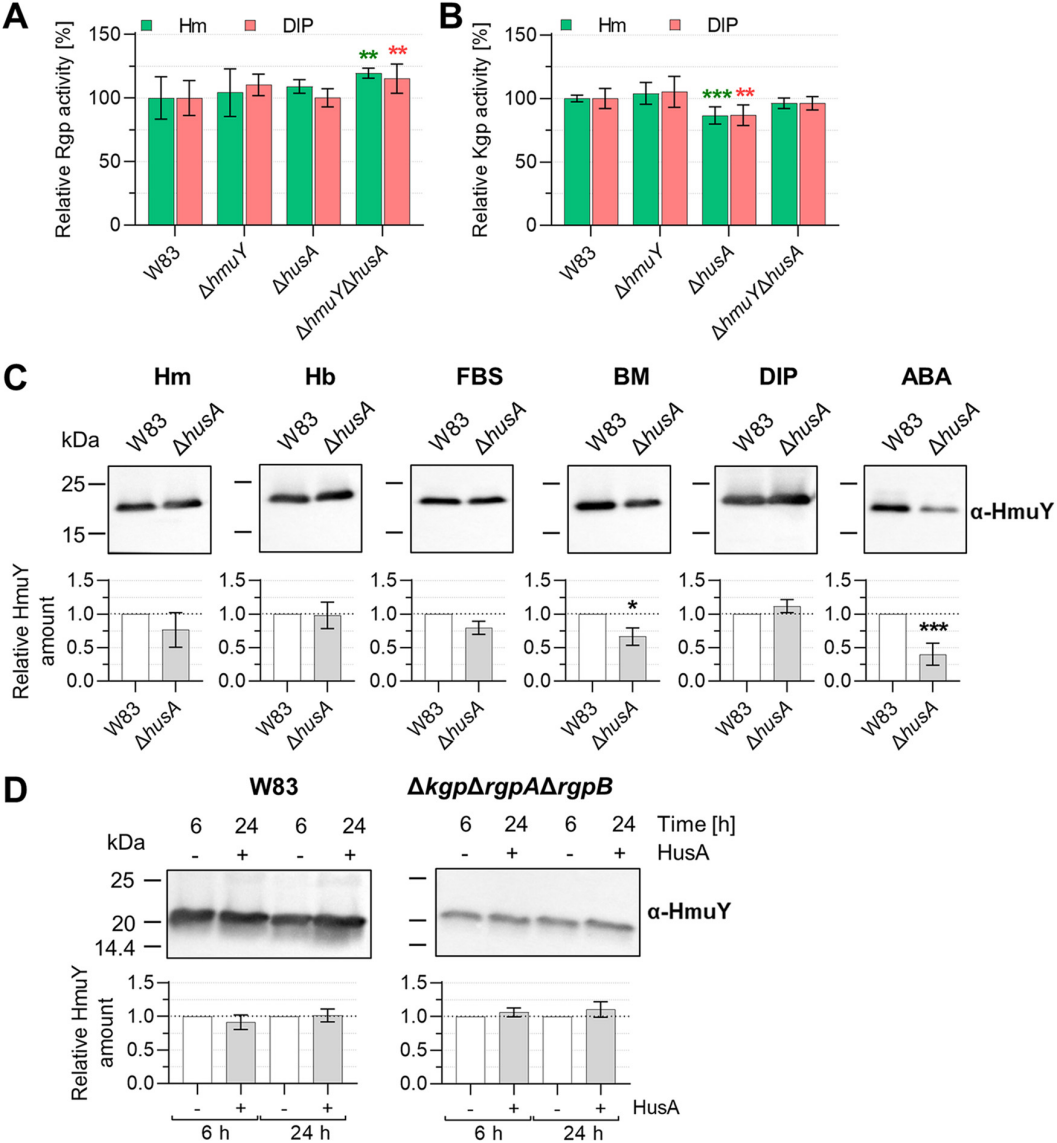
Influence of the lack of HmuY and HusA proteins on P. gingivalis heme acquisition. Rgp-specific (A) and Kgp-specific (B) proteolytic activities were determined in whole P. gingivalis cultures grown for 24 h in iron- and heme-rich conditions (Hm) or in iron- and heme-free conditions (DIP). Experiments were carried out three times in two biological replicates. (C) HmuY protein levels were examined in P. gingivalis cells grown for 24 h in basal medium (BM), BM supplemented with 7.7 μM heme (Hm), 2 μM hemoglobin (Hb), 5% fetal bovine serum (FBS), and 160 μM dipyridyl (DIP), or in P. gingivalis cells grown on anaerobic blood agar plates (ABA) for 5 days. (D) HmuY protein levels were also examined in whole P. gingivalis cultures grown in BM supplemented with 7.7 μM heme (Hm) alone or in this culture medium additionally supplemented with purified HusA protein at a final 5-μM protein concentration. The HmuY protein was detected using Western blotting and anti-HmuY antibodies (α-HmuY). Experiments were performed at least 3 times. *, *P < *0.05; **, *P < *0.01; ***, *P < *0.001.

The results presented above showed that the deletion of examined components of the main heme supply system in P. gingivalis influences its phenotype. Therefore, we examined whether hemophore-like proteins influence the production of other components engaged in heme acquisition. However, regardless of the conditions examined, deletion of the *hmuY* and/or *husA* gene did not affect production of transcripts encoding other examined genes, potentially involved in heme supply (Table S1). The only exception was a slightly increased *hmuR* transcript level in the Δ*hmuY* and Δ*hmuY* Δ*husA* strains (Table S1), all cultured in high-iron and -heme conditions. Such an effect could result from compensation for the lack of the HmuY protein by overproducing the HmuR protein, which could be sufficient to ensure the direct inflow of heme for bacteria under the conditions tested. This effect was not observed in the case of the *husB* gene, where only a slightly higher transcript level was observed in the Δ*husA* strain starved of iron and heme (Table S1). Additionally, no changes in expression of genes encoding gingipains and hemagglutinins were observed. Similar observations were made when specific Kgp and Rgp proteolytic activities were determined in the Δ*hmuY*, Δ*husA*, and Δ*hmuY* Δ*husA* strains in comparison to the wild-type W83 strain. Although statistically significant changes were found for some strains, they did not exceed 10 to 20% of the activity exhibited by the wild-type strain (Fig. 4A and B).

Although no changes in expression at the transcript level of the *hmuY* and *husA* genes were observed in the Δ*husA* and Δ*hmuY* strains when bacteria were cultured in Hm or DIP media, we aimed to examine whether HusA influences production of HmuY protein by P. gingivalis grown in conditions differing in heme and/or other iron sources. The mutant strain not producing HusA protein cultured in basal medium (BM) supplemented with heme (Hm), hemoglobin (Hb), or fetal bovine serum (FBS) produced similar amounts of the HmuY protein as the wild-type W83 strain (Fig. 4C). In contrast, a statistically significant difference was observed when the Δ*husA* strain was grown in heme-free but iron-containing BM, showing production of a lower HmuY protein amount (~70% of that found in the wild-type W83 strain). Surprisingly, this effect was not visible when bacteria were grown in iron- and heme-free medium (DIP). When bacteria were grown on ABA plates, where hemoglobin is the main heme source and bacteria efficiently produce virulence factors, including gingipains, a significant reduction in the HmuY protein production (~50%) was observed in the Δ*husA* strain (Fig. 4C).

**FIG 5:**
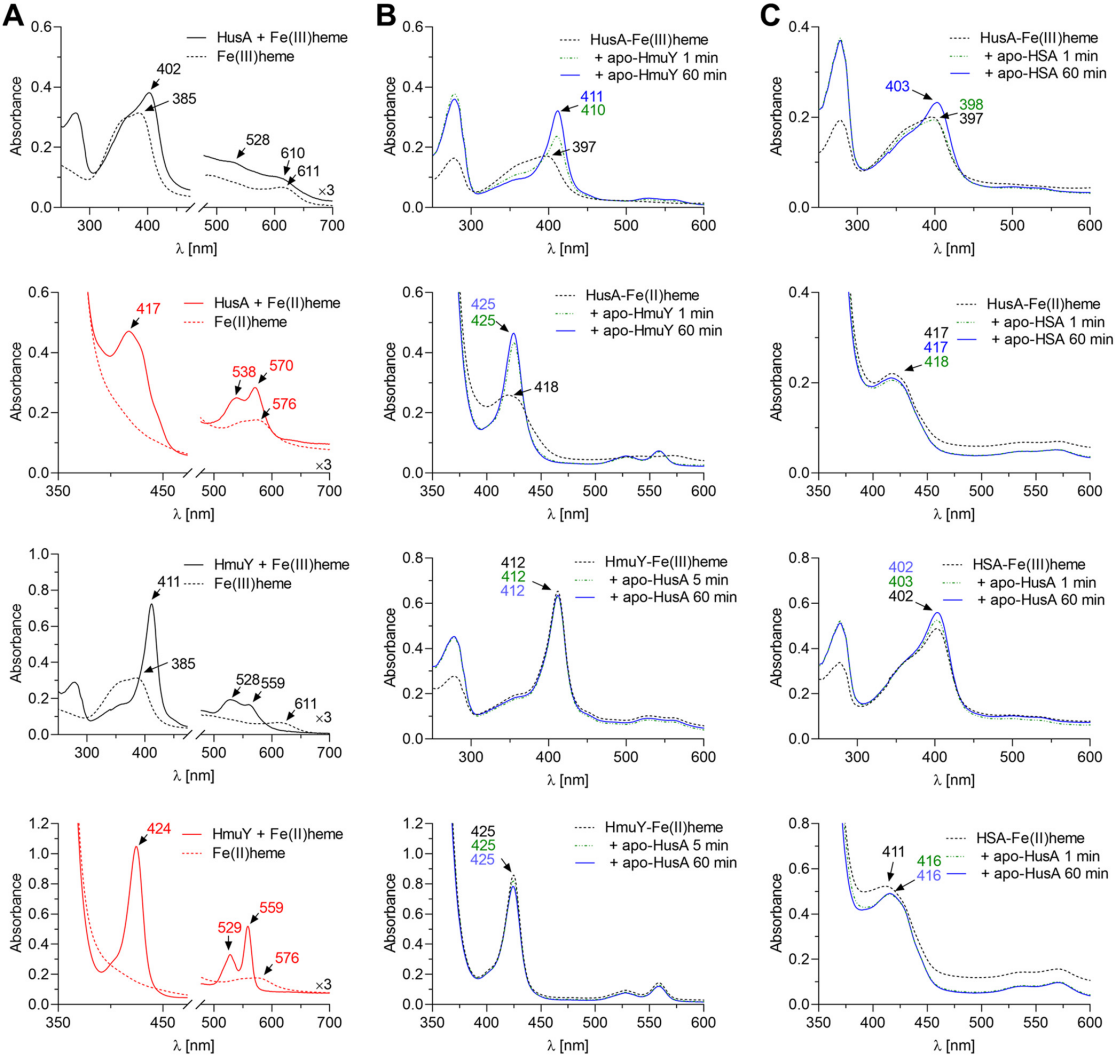
Analysis of heme sequestration. (A to C) Control UV-visible spectra of HusA-heme and HmuY-heme complexes (A), sequestration of heme from HusA-heme complex by apo-HmuY or from HmuY-heme complex by apo-HusA (B), and sequestration of heme from HusA-heme complex by apo-human serum albumin (HSA) or from HSA-heme complex by apo-HusA (C). Heme binding was examined under air (oxidizing) and reducing (addition of 10 mM sodium dithionite) conditions. UV-visible absorption spectra were recorded at the indicated time points after mixing of 5 μM holo-protein with 5 μM apo-protein.

According to previous findings (37, 38), we confirmed that HusA binds heme, but with significantly lower ability and in a different manner from HmuY (Fig. 5A). UV-visible absorbance spectra recorded for HmuY-heme complex under oxidizing or reducing conditions demonstrated sharp Soret maximum at 411 or 424 nm, respectively. Broad maxima at α- and β-bands, observed at 559 and 528 nm under oxidizing conditions, increased and became well resolved and sharp under reducing conditions. On the contrary, heme binding to HusA gave rise to a broad Soret maximum at 402 or 417 nm under oxidizing or reducing conditions, respectively. A broad Q-band maximum, observed under oxidizing conditions at 528 nm, under reducing conditions was transformed into two sharp Q-band maxima at 538 and 570 nm.

To explore the relationship between the two hemophore-like proteins in heme supply, we examined potential heme transfer occurring between HmuY and HusA. As shown in Fig. 5B, HmuY was able to efficiently capture heme bound to HusA under both oxidizing and reducing conditions. This effect was confirmed by quick change of the spectrum typical for the HusA-heme complex to that typical for the HmuY-heme complex (Fig. 5A). This was mostly visible by a rapid increase of the Soret maximum at 411 or 425 nm, confirming heme transfer from HusA to HmuY (Fig. 5B). We also demonstrated that HusA might capture heme bound to human serum albumin (HSA), but with low efficiency and only under reducing conditions. This process was confirmed by the shift of the Soret maximum from 411 nm to 416 nm, suggesting heme transfer from HSA to HusA (Fig. 5C).

Both HmuY and HusA proteins can be released from the outer membrane and exist in an extracellular environment in a soluble form (26, 38); therefore, we examined whether the addition of soluble HusA protein to the culture medium, thus mimicking overexpression of the *husA* gene, could facilitate the growth of P. gingivalis and influence HmuY production. Despite a high concentration (5 μM) of the purified HusA protein added to the culture medium, we did not detect any effect either on the growth of bacteria (data not shown) or on HmuY production (Fig. 4D). Moreover, electrophoretic analyses of proteins present in whole bacterial cultures showed that HusA protein was easily degraded by P. gingivalis proteases, whereas HmuY, as it has been shown before (26, 27, 59), was completely resistant (Fig. 4D and Fig. S2). Moreover, addition of 5 μM HusA to the cultures of the wild-type W83 and Δ*kgp* Δ*rgpA* Δ*rgpB* strains did not affect the production of HmuY (Fig. 4D). Therefore, we assumed that the soluble fraction of HusA does not influence production of HmuY, but the HusA amount could be regulated through its degradation by gingipains (Fig. S2).

### Importance of gingipains for P. gingivalis heme acquisition with engagement of hemophore-like proteins.

**FIG 6:**
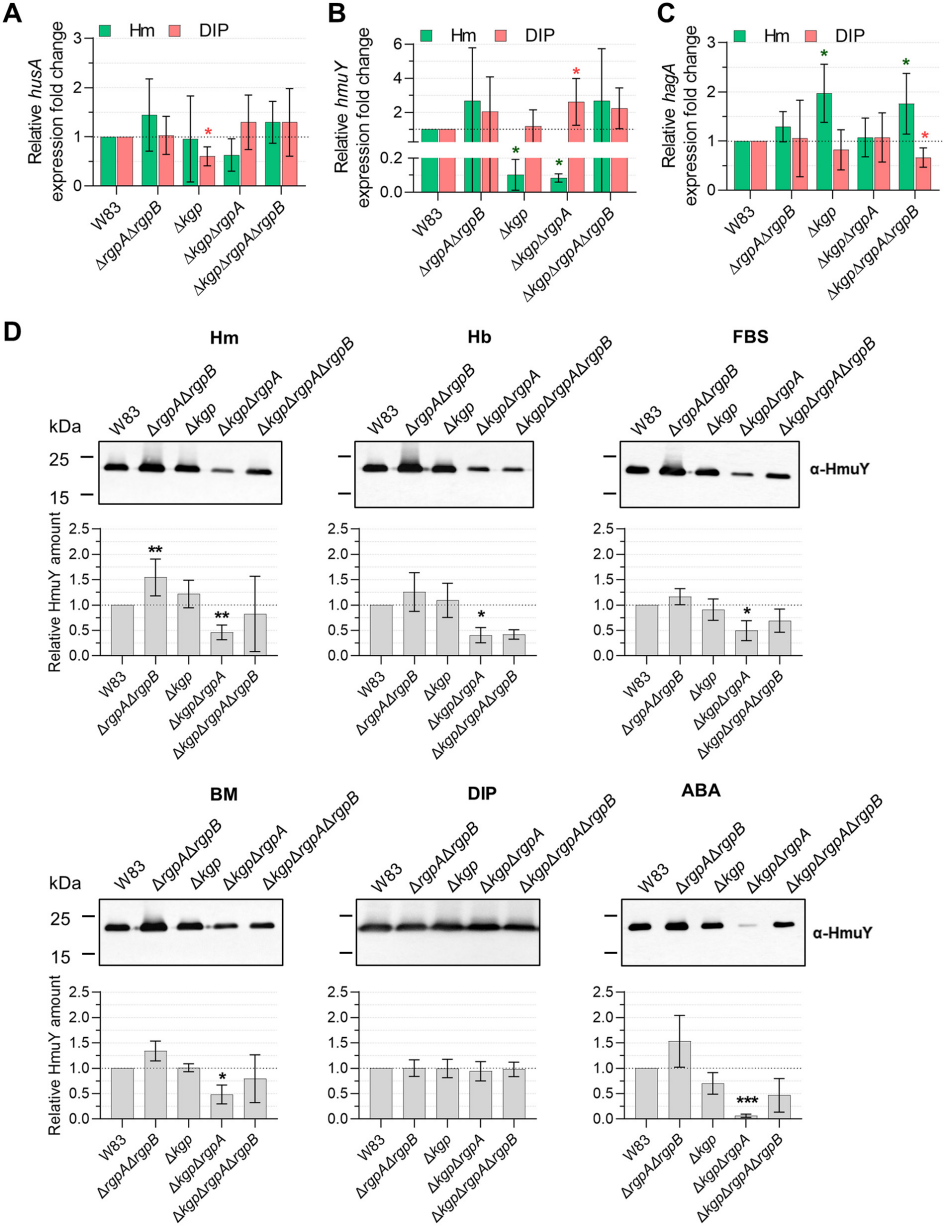
Influence of the lack of gingipains on P. gingivalis heme acquisition. Relative transcript levels of *husA* (A), *hmuY* (B), and *hagA* (C) were determined using RT-qPCR in P. gingivalis W83-derived gingipain mutant strains in comparison to the wild-type W83 strain grown for 24 h in iron- and heme-rich conditions (Hm) or in iron- and heme-depleted conditions (DIP). (D) HmuY levels were examined in P. gingivalis cells grown for 24 h in basal medium (BM), or BM supplemented with 7.7 μM heme (Hm), 2 μM hemoglobin (Hb), 5% fetal bovine serum (FBS), and 160 μM dipyridyl (DIP), or P. gingivalis cells grown on anaerobic blood agar plates (ABA) for 5 days. HmuY protein was detected using Western blotting and anti-HmuY antibodies (α-HmuY). *, *P* < 0.05; **, *P* < 0.01; ***, *P* < 0.001.

Gingipains, mainly Kgp, play an important role in heme supply due to their ability to degrade hemoproteins and release heme, which can be subsequently bound efficiently by HmuY and with low ability by HusA (Fig. 1A and Fig. 4A). Gingipains, mainly RgpA, also participate in hemoglobin oxidation, which facilitates heme sequestration from methemoglobin by HmuY (Fig. 1A). Therefore, we assumed that there might be a link between production of gingipains and proteins with hemophore-like function. To confirm this, we analyzed the influence of inactivation of gingipains on expression of *husA* and *hmuY* genes. The *husA* gene expression did not change significantly in gingipain mutant strains (Fig. 6A). A statistically significant, but very small, decrease in the *husA* transcript level was observed only in the Δ*kgp* strain starved of iron and heme. On the other hand, analysis of *hmuY* gene expression showed that Δ*kgp* and Δ*kgp* Δ*rgpA* strains grown in iron- and heme-rich conditions produced significantly smaller amounts of the *hmuY* transcript (Fig. 6B). Surprisingly, the Δ*kgp* Δ*rgpA* strain starved of iron and heme produced higher levels of mRNA encoding HmuY. To further analyze the HmuY protein production, P. gingivalis gingipain mutant strains were grown in liquid media differing in heme and/or iron availability (Hm, BM, DIP) and in liquid media supplemented with hemoglobin or FBS. HmuY protein production was significantly decreased during P. gingivalis growth in all but iron- and heme-depleted conditions (DIP) in the Δ*kgp* Δ*rgpA* strain and slightly decreased in the Δ*kgp* Δ*rgpA* Δ*rgpB* strain, where hemoglobin was the main heme source (Fig. 6D). Interestingly, analysis at the protein level showed that the Δ*rgpA* Δ*rgpB* strain produced slightly larger amounts of HmuY protein than the wild-type W83 strain grown in Hm medium (Fig. 6D), which is in agreement with transcript level (Fig. 6B). No significant changes in the HmuY production were observed when bacteria were grown in iron- and heme-free medium (DIP) (Fig. 6D), which is in good accordance with the *hmuY* transcript level (Fig. 6B). Similar analysis carried out using P. gingivalis grown on ABA plates generally confirmed the above-described findings, demonstrating significantly lower HmuY production in the Δ*kgp* Δ*rgpA* strain (Fig. 6D).

### Potential of mutual relationship in iron and heme homeostasis of HmuY, HusA, and gingipains.

**FIG 7:**
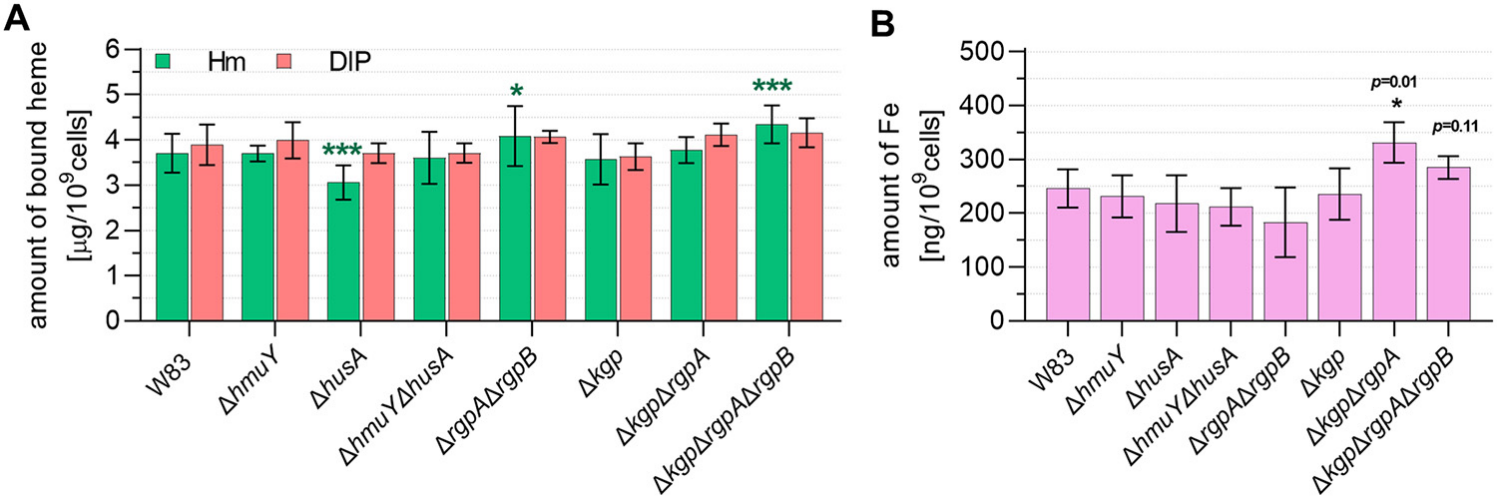
Influence of the lack of examined proteins on P. gingivalis iron and heme homeostasis. (A) The heme-binding ability was examined using bacteria cultured for 24 h in heme- and iron-repleted (Hm) or heme- and iron-depleted (DIP) conditions. (B) The intracellular amount of iron was measured in bacterial cells grown for 24 h in Hm medium using the colorimetric method based on Fe^2+^ chelator ferrozine. Results are shown as mean values ± standard deviation (mean ± SD) from at least three independent biological replicates of mutant strains versus the wild-type W83 strain. *, *P < *0.05; ***, *P < *0.001.

Many of the surface proteins produced by P. gingivalis (including gingipains, HmuY, and HusA) have the ability to bind heme, and many of them are produced in an iron- and heme-dependent manner (e.g., references 19, 30, 32, 37 to 39, 60, and 61); therefore, we analyzed how the tested mutations affected the ability of P. gingivalis cell surface macromolecules to bind heme. No differences between strains were observed when bacteria were grown in DIP medium (Fig. 7A); however, the Δ*husA* strain bound less heme than the wild-type W83 strain and other examined mutant strains when cultured in Hm medium. This may be caused directly by the lack of the HusA protein on the bacterial cell surface or by the indirect influence of the mutation on the bacterial phenotype. The Δ*rgpA* Δ*rgpB* and Δ*kgp* Δ*rgpA* Δ*rgpB* strains were able to bind slightly more heme than the wild-type W83 strain when grown in Hm medium. It could be suggested that because of disturbance in heme uptake, strains lacking RgpA and RgpB could overproduce other outer membrane proteins to compensate for heme binding. As shown in Fig. 6C, increased mRNA levels encoding *hagA* gene expression could be observed in the Δ*kgp* and Δ*kgp* Δ*rgpA* Δ*rgpB* strains, but only in bacteria cultured in Hm medium. We have suspected that overproduction of other proteins involved in heme uptake could, at least in part, explain the larger ability of heme binding by surface molecules of P. gingivalis, subsequent higher heme transport into the P. gingivalis cell, and higher iron availability. As shown in Fig. 7B, Δ*kgp* Δ*rgpA* and Δ*kgp* Δ*rgpA* Δ*rgpB* strains contained more intracellular iron than the other examined strains.

To understand this phenomenon, we further investigated how inactivation of certain gingipains influences the proteolytic activity of P. gingivalis in conditions used in our study. As expected, Arg-specific protease activity was not detected in Δ*rgpA* Δ*rgpB* and Δ*kgp* Δ*rgpA* Δ*rgpB* strains, whereas the Δ*kgp* Δ*rgpA* mutant strain exhibited decreased activity compared to the wild-type W83 strain (Fig. S3A). Also as expected, Lys-specific activity was not detected in all mutant strains with an inactivated *kgp* gene (Fig. S3B). Our results are consistent with the published data summed up in Tables S2 and S3. Some discrepancies observed could be explained by differences in culture conditions and strains used. Importantly, as shown in Fig. S3B and Table S3, the Δ*rgp*A Δ*rgpB* strain exhibited lower Kgp activity in conditions used in our studies. This effect might be caused by disturbed processing of Kgp in the absence of Rgp activity, which could result in the production of Kgp with reduced activity or in rapid degradation of the improperly processed enzyme. The activity, mainly that performed by Kgp gingipain, is also important for heme storage on the P. gingivalis surface (45, 62); therefore, the lack of pigment formation on the cell surface of the Δ*rgp*A Δ*rgpB* strain is understandable. To verify such a possibility, we examined gingipains at the protein level in whole P. gingivalis cell lysates. As expected, the Δ*rgpA* Δ*rgpB* strain contained large amounts of the pro-Kgp form (~180 kDa), which was not processed, and the mature form of the catalytic domain was not noticeable (~48 kDa) (Fig. S3C). Therefore, we performed complementation experiments, first using bacteria grown on ABA plates. When the Δ*rgp*A Δ*rgpB* strain was cocultured with the Δ*kgp* or Δ*kgp* Δ*rgpA* strain, where RgpA and/or RgpB activity was restored, the bacteria exhibited pigment formation, i.e., heme storage on the bacterial cell surface (Fig. 2B). This would suggest that Kgp activity was restored due to the proper Kgp processing. To further verify these findings, we performed complementation experiments in liquid cultures. For this purpose, we added a source of Arg-specific gingipains in the form of the Δ*kgp* or Δ*kgp* Δ*rgpA* strain, producing functional RgpA and/or RgpB. We found that the addition of bacterial cells containing RgpA and/or RgpB produced by Kgp mutant strains (Δ*kgp* or Δ*kgp* Δ*rgpA*) partially restored Kgp activity of the bacterial culture (Fig. S4A). Our results confirmed that complementation of RgpA and/or RgpB activity restored Kgp maturation and subsequent proteolytic activity (Fig. S4B).

## DISCUSSION

Several *in vitro* studies suggested that only some P. gingivalis strains, including the W83 strain or its variants, may have the capacity to cause dysbiosis in the oral cavity (60, 63–66). Differences in the ability to cause localized or systemic infections between strains have been proven in animal model studies (63, 67–70). To generate diversity between strains or among strains, P. gingivalis uses domain rearrangements and genetic exchange, mostly in genes encoding cell surface virulence factors, including those engaged in heme supply (64, 66, 71, 72). These differences may contribute to better adaptation to the changing conditions of the external environment, leading to higher virulence of some strains or variants. P. gingivalis used such mechanisms to evolve multiple mechanisms to efficiently acquire heme from the external environment, including expression of gingipains and Hmu and Hus systems (Fig. 1A).

Heme is a key compound that plays a crucial role in P. gingivalis virulence, as evidenced by the fact that expression of many genes is altered when the bacterium is starved of iron and heme (60, 61, 73–75). It is also worth mentioning that heme availability varies at different stages of P. gingivalis infection (Fig. 1B to D), which forces the rapid response of the bacterium to changing environmental conditions, resulting in differential expression of genes whose products are crucial for heme acquisition. It is well-known that genes encoding *hmu* and *hus* operons are expressed depending on the availability of iron and heme in the external environment, with *hmuY* expression increasing several hundred times (19). Moreover, *hmuY* and *husA* expression increases during the invasion of human cells (31, 38). Expression of *kgp*, *rgpA*, and *rgpB* is not strictly dependent on the availability of iron and heme but, rather, on bacterial growth phase and culture density (76–78). Importantly from the pathogenesis side of view, genes encoding gingipains and genes of the *hmu* operon are among the most upregulated genes of P. gingivalis examined in periodontitis patients (6).

Many studies have shown that gingipains are important for P. gingivalis virulence, and gingipain deletion mutant strains showed significantly decreased virulence in both *in vitro* cell cocultures and *in vivo* animal models (79–81). Results gained from P. gingivalis-THP-1-derived macrophages (31) or P. gingivalis-H413 oral mucosa epithelial cell (38) cocultures demonstrated that deletion of the *hmuY* or *husA* gene resulted in lower infection ability, as well as in lower intracellular viability inside host cells of the respective P. gingivalis mutant strains. In this study, we observed that the invasion ability of gingival keratinocytes was significantly lower when P. gingivalis did not produce both HmuY and HusA proteins. Taking into account the importance of the above-mentioned genes for P. gingivalis virulence, this study aimed to examine whether mutualism between gingipains and hemophore-like proteins might exist. Previous studies suggested that proteins composing the P. gingivalis Hmu system (mainly HmuY and HmuR) and gingipains can be synergistically involved in some stages of heme acquisition (35, 51, 53, 54, 77) (Fig. 1A). The results obtained in this study further confirmed that function of HmuY and Kgp/RgpA gingipains is correlated to ensure efficient heme supply, and the production of these proteins can be synergistically linked. We also found that the lack of active Kgp and RgpA may result in changes in the production of the HmuY protein. In contrast to RgpB, both Kgp and RgpA gingipains are composed of noncovalently attached proteolytic and hemagglutinin/adhesin domains (39), and their hemagglutinin domains are able to bind heme and hemoglobin (51). Therefore, the role of Kgp and RgpA may be of greater importance in heme acquisition than RgpB, the latter being more involved in degradation of other host proteins. The P. gingivalis strain lacking RgpA and Kgp activity grown in the presence of heme excess showed larger amounts of intracellular iron, which most likely resulted in the reduction of HmuY protein production. It has been reported by others that deletion of *hagB* and *hagC* genes resulted in increased expression of *hagA*, *rgpA*, and *kgp* genes, thus compensating for the lack of the HagB and HagC proteins (82). Therefore, based on our findings, one may suggest that the increase in the intracellular iron content could be explained by increased production of other proteins involved in heme binding, such as HagA, and subsequent heme transport through the outer membrane by HmuR, finally reducing *hmuY* gene expression. Another explanation could be overexpression of systems transporting nonheme iron, compensating for the lack of the examined proteins. P. gingivalis produces FeoB1 protein, which is responsible for the transport of iron (83). Due to the fact that P. gingivalis uses mainly heme as a source of iron, we believe that the Iht system and the FeoB1 could work simultaneously. One may hypothesize that IhtB acts as a reverse ferrochelatase that removes iron from heme and delivers it to IhtB for iron transport to the periplasm (19). Subsequently, iron could be transferred to the cytoplasm using IhtC-E proteins or the FeoB1 protein.

As observed by others (84) and in this study, gingipain activity is also important for gingipain maturation. Disruption of this process may result in inactive/inefficient mechanisms in which gingipains are involved. Since Kgp releases HmuY protein from the lipid anchor and digests hemoproteins to release heme and RgpA participates in oxidation of oxyhemoglobin to methemoglobin, both gingipains are important for the efficient functioning of the HmuY protein and the entire Hmu system. In addition, our study suggested that gingipains may be involved in regulation of availability of other heme-binding proteins, such as soluble HusA protein, due to their degradation.

It seems that cooperation in heme supply between gingipains and HmuY could be less important in the healthy periodontium and in the initial stages of the infection without bleeding (moderate periodontitis), where the amounts of iron and heme are limited. Under such conditions, HmuY is produced at higher levels and is able to capture heme directly from albumin (35), which is the normal front-line heme scavenger *in vivo*, and from hemopexin (27), both available from gingival crevicular fluid (Fig. 1B and C). It is worth mentioning here that HmuY binds heme with high affinity and is resistant to proteolytic activity, demonstrated by a variety of bacterial and host proteases (12, 26, 27, 59), which allows for its accumulation and spread. Therefore, HmuY produced in large amounts would be able to bind heme occurring locally in the vicinity of P. gingivalis, including that bound by other hemoproteins produced by P. gingivalis (including HusA). In addition, by spreading through the structures of the biofilm, HmuY could acquire heme from cohabitating bacteria, from which it can directly sequester heme or can bind heme released from hemoproteins by proteolysis (27, 59, 85).

Although gingipains and HmuY are encoded by the genes located in different regions of the P. gingivalis genome, their correlated production could be explained by a common regulatory system that works in P. gingivalis in a different manner from other Gram-negative bacteria. One example could be P. gingivalis ferric uptake regulator homolog (PgFur). Recently, we (60, 73, 86) and others (61, 87, 88) demonstrated that PgFur does not regulate iron homeostasis in a manner typical for classical Fur proteins by changing expression of genes involved in iron acquisition and storage in other Gram-negative bacteria (89). We have proposed that expression of proteins involved in iron and heme acquisition in P. gingivalis, namely, hemagglutinins, gingipains, and HmuY, could be regulated by PgFur, but in a different manner (60, 73, 86). According to our hypothesis, P. gingivalis employs PgFur to initiate a complex, multilayer network used to regulate virulence in the host. Another regulatory system responsible for this phenomenon could be the two-component HaeSR system, which has been shown to regulate the expression of *hmuY*, *kgp*, and *rgpA* genes (90).

In conclusion, our study further demonstrated that P. gingivalis is a unique pathogen that can efficiently modulate its behavior to adapt to the hostile host environment, both to proliferate in the complex microbiome consortium and to survive inside host cells, as well as in other niches of the host. The main findings of this study were that (i) heme acquisition in P. gingivalis is a complex process, in which mainly the Hmu system, with the leading role played by the HmuY hemophore-like protein, and Kgp/RgpA gingipains prefer cooperative interplay; (ii) the production of Kgp/RgpA gingipains and HmuY is related mainly when P. gingivalis grows in conditions rich in iron and heme sources, resembling those found in severe periodontitis; (iii) the only correlation in the production of HmuY and HusA hemophore-like proteins could occur in P. gingivalis grown on ABA plates, conditions rich in iron and heme sources and mimicking the environment typical for severe periodontitis; and (iv) HusA hemophore-like protein could be involved in other, so-far-uncharacterized iron and heme supply mechanisms.

## MATERIALS AND METHODS

### Bacterial strains and growth conditions.

P. gingivalis wild-type W83 strain, Δ*hmuY*, Δ*husA*, Δ*hmuY* Δ*husA*, Δ*kgp*, Δ*rgpA* Δ*rgpB*, Δ*kgp* Δ*rgpA*, and Δ*kgp* Δ*rgpA* Δ*rgpB* mutant strains constructed in the W83 wild-type strain (Table 1) were grown anaerobically (Whitley A35 anaerobic workstation; Bingley, UK) at 37°C for 5 days on anaerobic blood agar (ABA) plates composed of Schaedler broth (containing hemin and l-cysteine) and supplemented with 5% sheep blood and menadione (Argenta, Poznań, Poland). These cultures were inoculated into liquid basal medium (BM) prepared with 3% Trypticase soy broth (Becton, Dickinson, Sparks, MD, USA), 0.5% yeast extract (Biomaxima), 0.5 mg/L menadione (Fluka, Munich, Germany), and 0.05% l-cysteine (Sigma-Aldrich, St. Louis, MO, USA). To grow bacteria in high-iron and -heme conditions, BM medium was supplemented with 7.7 μM hemin chloride (Fluka) (Hm), 2 μM hemoglobin (Sigma-Aldrich, St. Louis, MO, USA) (Hb), or 5% fetal bovine serum (Sigma-Aldrich, St. Louis, MO, USA) (FBS), and to grow bacteria in low-heme conditions, no heme source was added (BM), or no heme was added and iron was chelated by addition of 160 μM 2,2-dipyridyl (Sigma-Aldrich) (DIP).

To monitor growth of P. gingivalis, bacteria were grown for 3 passages in Hm medium. Then, 200 μL of fresh Hm or DIP media was inoculated with bacteria at starting optical density at 600 nm (OD_600_) equal to 0.2, and the growth rate was measured using a Stratus plate reader (Cerillo, Charlottesville, VA, USA) every 2 h for 36 h.

### Construction of P. gingivalis mutant strains.

The Δ*hmuY* mutant strain was constructed by inactivation of the *hmuY* gene in the wild-type W83 strain (see Fig. S1 in the supplemental material). A DNA fragment encompassing the mutated region was amplified using genomic DNA isolated from the Δ*hmuY* mutant strain (TO4) previously constructed in the A7436 genetic background (29) and primers listed in Table S4. The linear DNA fragment was introduced into the W83 strain by electroporation (29). The mutant strain was selected on ABA plates in the presence of 5 μg/mL erythromycin. Homologous recombination between linear DNA construct and chromosomal DNA was monitored by PCR, DNA sequencing (Microsynth Seqlab GmbH, Gottingen, Germany), and Western blotting (Fig. S1) using anti-HmuY antibodies (GenScript USA Inc., Piscataway, NJ, USA), as described previously (75).

The Δ*husA* mutant strain was constructed by inactivation of the *husA* gene in the wild-type W83 strain (Fig. S1). The internal part of the *husA* gene was replaced with the tetracycline resistance cassette (*tetQ*) from Bacteroides thetaiotaomicron (GenBank accession number X58717) encoded on a pTC-COW vector (29, 91, 92). Flanking regions of the *husA* gene and *tetQ* gene were amplified using PCR and primers listed in Table S4. Obtained DNA sequences were ligated using NEBuilder HiFi DNA Assembly (New England Biolabs, Ipswich, MA, USA). The resulting DNA fragment was PCR amplified with respective primers (Table S4) and introduced into the W83 strain or Δ*hmuY* mutant strain by electroporation (29). Mutant strains were selected on ABA plates in the presence of 2 μg/mL tetracycline. Homologous recombination between constructs and chromosomal DNA was monitored by PCR, DNA sequencing (Microsynth Seqlab GmbH), and RT-PCR (Fig. S1).

To construct the Δ*hmuY* Δ*husA* mutant strain, the *husA* gene was inactivated in the Δ*hmuY* mutant strain as described above. The double mutant strain was selected on ABA plates in the presence of 5 μg/mL erythromycin and 2 μg/mL tetracycline.

### Overexpression and purification of proteins.

The modified pMAL-c5x_His plasmid constructed in our previous study (86) was used to clone the DNA sequence encoding the HmuY protein (GenBank accession number AKV63662), lacking the predicted signal peptide and five additional N-terminal amino acid residues not present in the soluble HmuY protein (MKKIIFSALCALPLIVSLTSCGKKK) or the DNA sequence encoding HusA protein (GenBank accession number AKV65255), lacking the predicted signal peptide (MKTFKRIALLLVAGFAGLCATSA), into XmnI and BamHI restriction sites using NEBuilder HiFi DNA Assembly (New England Biolabs). All primers are listed in Table S4. Proteins were overexpressed in Escherichia coli BL21-CodonPlus (DE3)-RIL (Agilent Technologies, Santa Clara, CA, USA) and purified from soluble fractions obtained from E. coli cell lysates with subsequent cutting off of the fusion His-maltose-binding protein (MBP) tag from the recombinant proteins according to the manufacturer’s protocol (New England Biolabs). The concentration of the purified proteins was determined spectrophotometrically using the empirical molar absorption coefficients (mM^−1^cm^−1^) of 36.68 for HmuY (25) and 33.81 for HusA (this study).

### Heme-protein complex formation.

Heme (hemin chloride; Fluka) solutions were prepared as reported previously (27). The formation of protein-heme complexes was examined in 20 mM sodium phosphate buffer, pH 7.4, containing 140 mM NaCl (phosphate-buffered saline [PBS]). UV-visible absorption spectra were recorded in the range of 250 to 700 nm with a double-beam Jasco V-750 spectrophotometer (Jasco GmbH, Pfungstadt, Germany) using samples prepared in PBS and cuvettes with 10 mm path length. To analyze the redox properties of the heme iron, 10 mM sodium dithionite (Sigma-Aldrich) prepared in PBS was used as the reductant.

### Heme sequestration experiments.

Protein-heme complexes were prepared in PBS by incubating a 120-μM stock solution of human serum albumin (HSA) (Sigma-Aldrich), HusA, or HmuY in PBS at 37°C with heme at a 1:1.2 protein/heme molar ratio and subsequently passed through Zeba Spin desalting columns (Thermo Fisher, Scientific, Waltham, MA, USA) to ensure that no uncomplexed heme remained. Coincubation of respective proteins was carried out in PBS at 22°C and monitored by UV-visible absorbance spectroscopy under oxidizing or reducing conditions using each protein at 5 μM concentration (27, 85).

### Sodium dodecyl sulfate-polyacrylamide gel electrophoresis and Western blotting.

For SDS-PAGE, samples derived from liquid bacterial cultures were prepared and standardized to the same OD_600_ and analyzed as reported previously (60, 75). To analyze proteins produced by bacteria grown on ABA plates, 20 μL of the overnight liquid bacterial cultures at OD_600_ equal to 2 was applied in a spot of ~1 cm diameter and cultured for 5 days. Then, bacteria were collected from ABA plates and suspended in 1 mL of PBS containing a protease inhibitor cocktail (Bimake, Houston, TX, USA) and stored at −80°C. After thawing, bacteria were lysed by sonication (Sonopuls HD 4100; Bandelin, Berlin, Germany). The protein concentration in the bacterial cell lysates was determined with Roti Nanoquant (Carl Roth, Karlsruhe, Germany). Proteins present in bacterial cell lysates (2.5 μg) were separated using SDS-PAGE and stained with Coomassie brilliant blue G-250 (Coomassie brilliant blue [CBB]).

For Western blotting, samples were separated by SDS-PAGE, transferred onto nitrocellulose membranes (Millipore, Billerica, MA, USA), probed with rabbit anti-HmuY antibodies (1:10,000 to 1:20,000; GenScript USA Inc.), rabbit anti-Kgp catalytic domain antibodies (0.5 μg/mL; Cusabio, Houston, TX, USA), or rabbit anti-RgpB catalytic domain antibodies (0.5 μg/mL; Cusabio), and complexes formed were detected using goat horseradish peroxidase (HRP)-conjugated anti-rabbit IgG antibodies (1:20,000; Sigma-Aldrich), chemiluminescence staining (PerkinElmer, Waltham, MA, USA), and the ChemiDoc imaging system (Bio-Rad Laboratories, Hercules, CA, USA) (65, 76). The relative amount of HmuY in the mutant strains compared to the wild-type strain was evaluated by band densitometric analysis (relative quantification) using Image Lab 6.0.1 software (Bio-Rad).

### Determination of heme binding by P. gingivalis.

Analysis of heme binding by P. gingivalis cells was performed as described before (51) with minor modifications. Briefly, overnight bacterial cultures (20 mL) were centrifuged (4,000 × *g*, 30 min, 4°C) and washed twice with PBS. Bacterial suspensions (800 μL) at OD_600_ equal to 1.25 were mixed with 200 μL of 50 μg/mL hemin chloride solution diluted in PBS and incubated for 1 h at 37°C. Then, samples were centrifuged (10,000 × *g*, 10 min, room temperature), and the supernatant’s absorbance at 385 nm was measured. Control samples included all components, excluding bacteria. The difference in absorbance values between the control and the test samples indicated the amount of heme bound by bacteria.

### Determination of intracellular iron content.

Overnight bacterial cultures (20 mL) were centrifuged (4,000 × *g*, 30 min, 4°C) and washed twice with PBS. Bacterial suspensions (4 mL) at OD_600_ equal to 2 were lysed by sonication. For determination of intracellular iron, an iron assay kit was used according to the manufacturer’s protocol (Sigma-Aldrich). Alternatively, a ferrozine-based method was used (93). Briefly, 15 μL of 10% SDS (Carl Roth) was added to 735 μL of the lysed bacterial samples, and samples were incubated for 15 min at 37°C. Then, to reduce all Fe ions, 35 μL of 1% ascorbic acid (Carl Roth) was added, and samples were incubated for 30 min at room temperature. Afterward, 250 μL of 5.35 mM ferrozine [3-(2-pyridyl)-5,6-diphenyl-1,2,4-triazine-*p*,*p*′-disulfonic acid monosodium salt hydrate; Sigma-Aldrich] solution in 32.9% (vol/vol) acetic acid (Carl Roth), containing 6.9% (vol/vol) ammonia (Carl Roth), was added. Samples were incubated for 60 min at room temperature and subsequently centrifuged (15,000 × *g*, 5 min, room temperature). The absorbance of the supernatants was measured at 562 nm, the wavelength corresponding to the maximum of the ferrozine complex with Fe(II). To determine the amount of iron in the sample, a standard curve was prepared using FeCl_3_ as a standard (Sigma-Aldrich) and treated as the bacterial samples.

### Determination of proteolytic activity.

Whole P. gingivalis cultures were examined in order to determine proteolytic activity specific for Arg- and Lys-specific gingipains. For this purpose, *N*α-benzoyl-d,l-arginine 4-nitroanilide hydrochloride (BApNA; Sigma-Aldrich) or *N*-(*p*-tosyl)-Gly-Pro-Lys 4-nitroanilide acetate salt (Sigma-Aldrich), respectively, was used as a substrate (60, 94).

### Phenotypic complementation of the Δ*rgpA* Δ*rgpB* mutant strain.

To complement the lack of Arg-specific gingipain activity, the Δ*rgpA* Δ*rgpB* strain was mixed with respective gingipain mutant strains. For this purpose, overnight cultures were used to inoculate fresh Hm medium to obtain bacterial cultures at an initial OD_600_ equal to 0.2 or a mixture of the Δ*rgpA* Δ*rgpB* strain with the Δ*kgp*, Δ*kgp* Δ*rgpA*, or Δ*kgp* Δ*rgpA* Δ*rgpB* strain at a ratio of 3:1 (vol/vol).

Complementation on ABA plates was performed by mixing overnight P. gingivalis cultures at a 1:1 (vol/vol) ratio. Mixed overnight liquid bacterial cultures (20 μL) were applied to the plate and cultured for 5 days.

### Reverse transcriptase quantitative PCR.

RNA was isolated from P. gingivalis cells and purified as described previously (60). A reverse transcriptase (RT) reaction was carried out using 500 ng of total RNA and a LunaScript RT supermix kit (New England Biolabs). Quantitative PCR (qPCR) was performed using a SensiFast SYBR No-ROX kit (Bioline, London, UK) and the LightCycler 96 system (Roche, Basel, Switzerland). The PCR was carried out as follows: initial denaturation at 95°C for 2 min, 35 to 40 cycles of denaturation at 95°C for 5 s, primer annealing at 60°C for 10 s, and extension at 72°C for 20 s. The melting curves were analyzed to monitor the quality of PCR products. Relative quantification of genes was determined in comparison to the P. gingivalis *16S rRNA* gene (gene ID 2552647) as a reference, using the threshold cycle (ΔΔ*Ct*) method. All primers used in this study are listed in Table S4. All samples and controls were run in triplicate in three independent experiments for the target and reference genes.

### Infection assay.

Immortalized human gingival keratinocytes (Gie-No3B11; ABM, Richmond, British Columbia, CA, USA) were used as an epithelial infection cell model. The cells were grown as described previously (17) in TM-040 medium (ABM), supplemented with 2% heat-inactivated fetal bovine serum (FBS; Cytogen, Zgierz, Poland), 2 mM l-glutamine (Cytogen), 100 U/mL penicillin, and 100 μg/mL streptomycin (Cytogen) in a humidified atmosphere of 95% air and 5% CO_2_ at 37°C in a CO_2_ incubator (Panasonic Healthcare Co. Ltd., Sakata, Oizumi-Machi, Ora-Gun Gunma, Japan). For the infection assay, 24-well plates (Corning, NY, USA) were used, and 1.0 × 10^4^ cells were seeded per well and cultured for 24 h. Then, the cells were washed 3 times with PBS, and fresh Dulbecco’s modified Eagle medium (DMEM) (Sigma-Aldrich) was added.

P. gingivalis was grown to the early stationary phase in Hm medium, centrifuged (4,000 × *g*, 20 min, 4°C), washed twice with PBS, and used for infection of keratinocytes with a multiplicity of infection (MOI) of 100. Keratinocytes and P. gingivalis cocultures were incubated for 4 h at 37°C in a humidified atmosphere of 95% air and 5% CO_2_ at 37°C in a CO_2_ incubator (Panasonic Healthcare Co. Ltd.). The medium was collected at time 0 and 4 h after infection, and serial dilutions were spread on ABA plates to determine the number of live bacteria that served as a control for repeatability of infection between strains. After 4 h incubation, to determine the number of live bacteria attached to and present inside keratinocytes, one portion of the cells was incubated in fresh DMEM medium. To determine the number of live bacteria inside keratinocytes only, the second portion of the cells was incubated in fresh DMEM medium supplemented with 300 μg/mL gentamicin (Sigma-Aldrich) and 200 μg/mL metronidazole (Fluka) to kill external bacteria. After 1 h incubation, cells were washed twice with PBS and lysed with sterile distilled water. Serial dilutions of the lysates were plated on ABA plates, and CFU per milliliter were determined. The experiment was carried out three times using two independent biological samples, each sample examined in two technical repetitions.

### Statistical analysis.

Statistical analysis was performed using unpaired Student's *t* test. When necessary, data were analyzed using one-way analysis of variance (ANOVA) with *post hoc* Tukey’s test. All results are shown as mean ± standard deviation (mean ± SD) or mean ± standard error (mean ± SE). For statistical analysis, GraphPad software (GraphPad Prism 8.0 Inc., San Diego, CA, USA) was used.

### Bioinformatics analyses.

The structures of HusA (PBD accession number 6CRL) and HmuY (PDB accession number 6EWM) proteins, as well as the structure models obtained from AlphaFold (95, 96) for the HusB (UniProt accession number Q7MSY4) and HmuR (UniProt accession number Q7MUG9) proteins were visualized using the Swiss-PdbViewer (97).
