# Supplementary material for: Interplay between Porphyromonas gingivalis Hemophore-Like Protein HmuY and Kgp/RgpA Gingipains Plays a Superior Role in Heme Supply
Source: Microbiol Spectr. 2023 Feb 8;11(2):e04593-22. doi: 10.1128/spectrum.04593-22 (PMC10100897; doi:10.1128/spectrum.04593-22)
Supplement: Supplemental File 1 — Supplemental material. Download spectrum.04593-22-s0001.pdf, PDF file, 0.7 MB [file spectrum.04593-22-s0001.pdf]

## SUPPLEMENTAL MATERIAL

**TABLE S1** Analysis of gene expression. Relative transcript levels determined using RT-qPCR in W83-derived Δ*hmuY*, Δ*husA* and Δ*hmuY*Δ*husA* mutant strains in comparison to the wild type W83 *P. gingivalis* strain grown for 24 hours in iron/heme rich conditions (Hm) or in iron/heme-depleted conditions (DIP). Positive/negative values indicate respectively increase/decrease in gene expression. Experiments were carried out two times in at least two biological replicates and results are shown as mean values±standard deviation (mean±SD).

| Strain | Δ*hmuY* versus W83 | | Δ*husA* versus W83 | | Δ*hmuY*Δ*husA* versus W83 | |
|---|---|---|---|---|---|---|
| Gene \ Conditions | Hm | DIP | Hm | DIP | Hm | DIP |
| *hmuY* | ND | ND | 1.14 ± 0.40 | 1.16 ± 0.50 | ND | ND |
| *husA* | -1.17 ± 0.26 | 1.11 ± 0.46 | ND | ND | ND | ND |
| *hmuR* | 1.88 ± 1.34 | -1.39 ± 1.14 | 1.21 ± 0.27 | 1.18 ± 0.61 | 1.55 ± 0.76 | -1.07 ± 0.84 |
| *husB* | -1.05 ± 0.27 | 1.23 ± 0.59 | 1.08 ± 0.45 | 2.65 ± 1.29 | -1.39 ± 0.20 | -1.07 ± 0.39 |
| *hbp35* | 1.35 ± 0.11 | 1.03 ± 0.16 | -1.27 ± 0.54 | 1.16 ± 0.39 | 1.38 ± 0.20 | 1.26 ± 0.13 |
| *pgdps* | -1.31 ± 0.14 | -1.06 ± 0.41 | -1.09 ± 0.21 | 1.87 ± 0.59 | -1.27 ± 0.15 | -1.25 ± 0.18 |
| *hagA* | 1.14 ± 0.10 | 1.05 ± 0.28 | -1.38 ± 0.48 | 1.53 ± 0.62 | 1.13 ± 0.13 | -1.15 ± 0.19 |
| *rgpA* | -1.03 ± 0.26 | 1.41 ± 1.00 | 1.12 ± 0.30 | -1.17 ± 0.38 | 1.06 ± 0.21 | 1.71 ± 1.07 |
| *rgpB* | -1.12 ± 0.41 | 1.04 ± 0.31 | 1.25 ± 0.30 | 1.10 ± 0.38 | 1.09 ± 0.56 | 1.23 ± 0.25 |
| *kgp* | -1.71 ± 1.08 | 1.20 ± 0.73 | 1.14 ± 0.78 | -1.08 ± 0.44 | -1.28 ± 1.01 | 1.81 ± 0.92 |

ND – transcript not detected

**TABLE S2** Comparative analysis of relative Rgp activity in gingipain mutant strains.

| Reference | This study | (1) | (2) | (3) | (4) | (5) | (6) | (7) |
|---|---|---|---|---|---|---|---|---|
| Strain | W83 | W50 | ATCC 33277 | A7436 | ATCC 33277 | W83 | W83 | W50 |
| Culture fraction | whole culture | whole culture | whole culture | whole culture | cell extract | whole culture | whole culture | whole culture |
| Medium | BM+Hm | BHI | Blood plates | Anaerobe Broth MIC | BHI | BHI | TSB | BHI |
| Culture phase | early stationary | early stationary | 48h | early stationary | stationary | expotential | early stationary | early stationary |
| WT | 100% | 100% | 100% | 100% | 100% | 100% | 100% | 100% |
| Δ*rgpA* | NT | ~50% | ~55% | NT | NT | 40% | ~45% | NT |
| Δ*rgpB* | NT | ~50% | ~52% | NT | NT | 60% | NT | NT |
| Δ*rgpA*Δ*rgpB* | 0% | 0-2% | 0% | NT | 0% | 2% | NT | 0% |
| Δ*kgp* | 106% | 100% | NT | 114% | 122% | 100% | ~100% | ~100% |
| Δ*kgp*Δ*rgpA* | 85% | NT | NT | NT | 93% | 46% | ~45% | NT |
| Δ*kgp*Δ*rgpB* | NT | NT | NT | NT | NT | 47% | NT | NT |
| Δ*kgp*Δ*rgpA*Δ*rgpB* | 0% | NT | NT | NT | 0% | NT | 0% | NT |

NT – not tested; BM, basal medium; BHI, brain heart infusion medium; TSB, tryptic soy broth; Hm, hemin; MIC, minimal inhibitory concentration

1. Aduse-Opoku J, Davies N, Gallagher A, Hashim A, Evans H, Rangarajan M, Slaney J, Curtis M. 2012. Generation of Lys-gingipain protease activity in *Porphyromonas gingivalis* W50 is independent of Arg-gingipain protease activities. Microbiology 146:1933-1940.
2. Chen T, Nakayama K, Belliveau L, Duncan MJ. 2001. *Porphyromonas gingivalis* gingipains and adhesion to epithelial cells. Infect Immun 69:3048-56.
3. Simpson W, Olczak T, Genco CA. 2004. Lysine-specific gingipain K and heme/hemoglobin receptor HmuR are involved in heme utilization in *Porphyromonas gingivalis*. Acta Biochim Pol 51:253-262.
4. Shi Y, Ratnayake D, Okamoto K, Abe N, Yamamoto K, Nakayama K. 1999. Genetic analyses of proteolysis, hemoglobin binding, and hemagglutination of *Porphyromonas gingivalis*. Construction of mutants with a combination of *rgpA*, *rgpB*, *kgp*, and *hagA*. J Biol Chem 274:17955-17960.
5. Dou Y, Robles A, Roy F, Aruni A, Sandberg L, Nothnagel E, Fletcher H. 2015. The roles of RgpB and Kgp in late onset gingipain activity in the *vimA*-defective mutant of *Porphyromonas gingivalis* W83. Mol Oral Microbiol 30:347-360.
6. Veillard F, Potempa B, Poreba M, Drag M, Potempa J. 2012. Gingipain aminopeptidase activities in *Porphyromonas gingivalis*. Biol Chem 393:1471–1476.
7. Rangarajan M, Aduse-Opoku J, Paramonov NA, Hashim A, Curtis MA. 2017. Hemin binding by *Porphyromonas gingivalis* strains is dependent on the presence of A-LPS. Mol Oral Microbiol. 32:365-374.

**TABLE S3** Comparative analysis of relative Kgp activity in gingipain mutant strains.

| Reference | This study | (1) | (2) | (3) | (4) | (5) | (6) | (7) |
|---|---|---|---|---|---|---|---|---|
| Strain | W83 | W50 | ATCC 33277 | A7436 | ATCC 33277 | W83 | W83 | W50 |
| Culture fraction | whole culture | whole culture | whole culture | whole culture | cell extract | whole culture | whole culture | whole culture |
| Medium | BM+Hm | BHI | Blood plates | Anaerobe Broth MIC | BHI | BHI | TSB | BHI |
| Culture phase | early stationary | early stationary | 48h | early stationary | stationary | expotential | early stationary | early stationary |
| WT | 100% | 100% | 100% | 100% | 100% | 100% | 100% | 100% |
| Δ*rgpA* | NT | 100% | ~100% | NT | NT | 100% | 100% | NT |
| Δ*rgpB* | NT | 100% | ~93% | NT | NT | 86% | NT | NT |
| Δ*rgpA*Δ*rgpB* | 7% | 50-100% | ~54% | NT | 30-146% | 100% | ND | ~40% |
| Δ*kgp* | 2% | 0-2% | NT | 6% | 0% | 0% | 0% | 0% |
| Δ*kgp*Δ*rgpA* | 2% | NT | NT | NT | 0% | 2% | 0% | NT |
| Δ*kgp*Δ*rgpB* | NT | NT | NT | NT | NT | 0% | NT | NT |
| Δ*kgp*Δ*rgpA*Δ*rgpB* | 0% | NT | NT | NT | 0% | NT | 0% | NT |

NT – not tested; BM, basal medium; BHI, brain heart infusion medium; TSB, tryptic soy broth; Hm, hemin; MIC, minimal inhibitory concentration


1. Aduse-Opoku J, Davies N, Gallagher A, Hashim A, Evans H, Rangarajan M, Slaney J, Curtis M. 2012. Generation of Lys-gingipain protease activity in *Porphyromonas gingivalis* W50 is independent of Arg-gingipain protease activities. Microbiology 146:1933-1940.
2. Chen T, Nakayama K, Belliveau L, Duncan MJ. 2001. *Porphyromonas gingivalis* gingipains and adhesion to epithelial cells. Infect Immun 69:3048-56.
3. Simpson W, Olczak T, Genco CA. 2004. Lysine-specific gingipain K and heme/hemoglobin receptor HmuR are involved in heme utilization in *Porphyromonas gingivalis*. Acta Biochim Pol 51:253-262.
4. Shi Y, Ratnayake D, Okamoto K, Abe N, Yamamoto K, Nakayama K. 1999. Genetic analyses of proteolysis, hemoglobin binding, and hemagglutination of *Porphyromonas gingivalis*. Construction of mutants with a combination of *rgpA*, *rgpB*, *kgp*, and *hagA*. J Biol Chem 274:17955-17960.
5. Dou Y, Robles A, Roy F, Aruni A, Sandberg L, Nothnagel E, Fletcher H. 2015. The roles of RgpB and Kgp in late onset gingipain activity in the *vimA*-defective mutant of *Porphyromonas gingivalis* W83. Mol Oral Microbiol 30:347-360.
6. Veillard F, Potempa B, Poreba M, Drag M, Potempa J. 2012. Gingipain aminopeptidase activities in *Porphyromonas gingivalis*. Biol Chem 393:1471–1476.
7. Rangarajan M, Aduse-Opoku J, Paramonov NA, Hashim A, Curtis MA. 2017. Hemin binding by *Porphyromonas gingivalis* strains is dependent on the presence of A-LPS. Mol Oral Microbiol. 32:365-374.

**TABLE S4** Primers used in this study.

| Primer name | 5′→3 ′ DNA sequence | Locus ID and/or gene abbreviation | Description (references) |
|---|---|---|---|
| F_hmuY_mut | cctgtttattgagcaaaggtctc | *hmuY* (*PG_RS06840*), *hmuR* (*PG_RS06845*), *ermF*, 3'DNA fragment flanking *hmuR* | Amplify DNA fragment used to generate Δ*hmuY* mutant strain in the wild type W83 strain (this study) |
| R_hmuY_mut | ttgccgaagaagaggttg | | |
| F1_mut_husA | tactacctctcccatggcattg | *husA* (*PG_RS09910*) | Amplify DNA fragments including *husA* gene flanking sequences and *tetQ* cassette for generation of the Δ*husA* mutant strain in the wild type W83 and Δ*hmuY* mutant strain (this study) |
| R1_mut_husA | gagatagaagcattagaacttggcacctt gtgcggatgttgcac | | |
| F3_mut_husA | cgtacgttaaggagataattcgttgtagca gagagcagccgacag | | |
| R3_mut_husA | cgaaagccgaccaaggcg | | |
| F2_mut_husA | gtgcaacatccgcacaaggtgccaagtt ctaatgcttctatctc | *tetQ* | |
| R2_mut_husA | ctgtcggctgctctctgctacaacgaatta tctccttaacgtacg | | |
| F1_husA_control | ccgagtcggctattatcctac | *husA* (*PG_RS09910*) | Primers used to verify the generation of Δ*husA* mutant strain in the wild type W83 and Δ*hmuY* mutant strains (this study) |
| F2_husA_control | tgccaagttctaatgcttctatctc | | |
| R_husA_control | ccgagtcggctattatcctac | | |
| F_HmuY_pMAL | ctcgggatcgagggaaggatggacgag ccgaaccaac | *hmuY* (*PG_RS06840*) | Amplify the *hmuY* gene used to clone into XmnI and BamHI restriction sites of pMAL-c5x_His plasmid (1) for the HmuY protein overexpression and purification (this study) |
| R_HmuY_pMAL | ctgcagggaattcggatccttatttaacgg ggtatgtataagtgaaag | | |
| F_HusA_pMAL | ctcgggatcgagggaaggcaagggacg gcttatgcc | *husA* *(PG_RS09910)* | Amplify the *husA* gene used to clone into XmnI and BamHI restriction sites of pMAL-c5x_His plasmid (1) for the HusA protein overexpression and purification (this study) |
| R_HusA_pMAL | ctgcagggaattcggatcctcactcttgg gtttctgccttg | | |
| HYq4_F | gcttcgaaatacgaaacgtg | *hmuY* (*PG_RS06840*) | Amplify fragment of the gene for RT-qPCR analysis (2) |
| HYq4_R | tatatccgtctgtcggaacg | | |
| 16SrRNA-F | cttgacttcagtggcggcag | *16S rRNA* (*PG_RS09195*) | Amplify fragment of the gene for RT-qPCR analysis (3) |
| 16SrRNA-R | agggaagacggttttcacca | | |
| rtHagA2F | aggtgtacttggcattccgtc | *hagA* (*PG_RS08090*) | Amplify fragment of the gene for RT-qPCR analysis (4) |
| rtHagA2R | cgtgtacgtgtagtcgttgga | | |
| F_husA qPCR | atcggctatgcgaagaagc | *husA* (*PG_ RS09910*) | Amplify fragment of the gene for RT-qPCR analysis (this study) |
| R_husA qPCR | gaagtaggctcgcggattag | | |
| F_hmuR_qPCR | ctaccgacaccatcgtatcc | *hmuR* (*PG_RS06845*) | Amplify fragment of the gene for RT-qPCR analysis (Smiga et al. 2019b) |
| R_hmuR_qPCR | cattgagctgatctctggaac | | |
| F_husB_qPCR | ggacgatatggaacccgact | *husB* (*PG_RS09905*) | Amplify fragment of the gene for RT-qPCR analysis (this study) |
| R_husB_qPCR | agccatcccgaatccatagg | | |
| F_kgp_qPCR | gagtgtgggtgctaatgccg | *kgp* (*PG_RS08105*) | Amplify fragment of the gene for RT-qPCR analysis (5) |
| R_kgp_qPCR | caccaatatgggtaatattgccg | | |
| F_rgpA_qPCR | cgcttcccattctatcacgc | *rgpA* (*PG_RS08940*) | Amplify fragment of the gene for RT-qPCR analysis (5) |
| R_rgpA_qPCR | cggatcttcgttacgcataatcat | | |
| F_rgpB_qPCR | aatgataagccttatactgtagctg | *rgpB* (*PG_RS02240*) | Amplify fragment of the gene for RT-qPCR analysis (5) |
| R_rgpB_qPCR | gttttgtgcttcgaataccatgc | | |
| F_pgdps_qPCR | aatatccgtggcgcagag | *pgdps* (*PG_RS00405*) | Amplify fragment of the gene for RT-qPCR analysis (this study) |
| R_pgdps_qPCR | cgagttcgtgctcttccttc | | |
| F_hbp35_qPCR | gaatggtgcggttactgtcc | *hbp35* (*PG_RS02720*) | Amplify fragment of the gene for RT-qPCR analysis (this study) |
| R_hbp35_qPCR | Gaatgcccaatgtttgatcc | | |

1. Smiga M, Bielecki M, Olczak M, Olczak T. 2019. *Porphyromonas gingivalis* PgFur is a member of a novel Fur subfamily with non-canonical function. Front Cell Infect Microbiol 9:233.
2. Gmiterek A, Wojtowicz H, Mackiewicz P, Radwan-Oczko M, Kantorowicz M, Chomyszyn-Gajewska M, Fraszczak M, Bielecki M, Olczak M, Olczak T. 2013. The unique *hmuY* gene sequence as a specific marker of *Porphyromonas gingivalis*. PLoS One 8(7):e67719.
3. Maeda H, Fujimoto C, Haruki Y, Maeda T, Kokeguchi S, Petelin M, Arai H, Tanimoto I, Nishimura F, Takashiba S. 2003. Quantitative real-time PCR using TaqMan and SYBR Green for *Actinobacillus actinomycetemcomitans*, *Porphyromonas gingivalis*, *Prevotella intermedia*, *tetQ* gene and total bacteria. FEMS Immunol Med Microbiol 39:81-86.
4. Ciuraszkiewicz J, Smiga M, Mackiewicz P, Gmiterek A, Bielecki M, Olczak M, Olczak T. 2014. Fur homolog regulates *Porphyromonas gingivalis* virulence under low-iron/heme conditions through a complex regulatory network. Mol Oral Microbiol 29:333-353.
5. Smiga M, Stepien P, Olczak M, Olczak T. 2019. PgFur participates differentially in expression of virulence factors in more virulent A7436 and less virulent ATCC 33277 *Porphyromonas gingivalis* strains. BMC Microbiol 19(1):127.

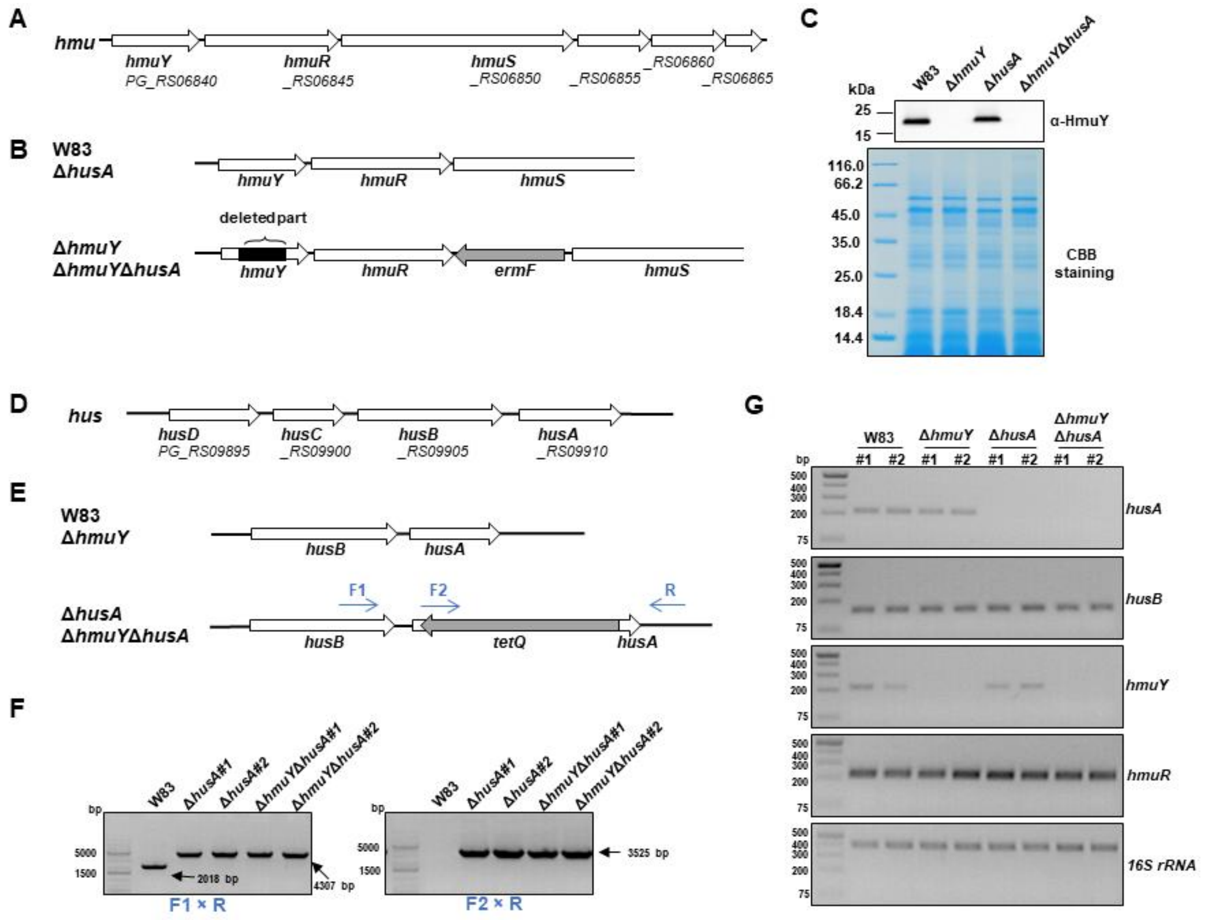


**FIG S1** Construction of mutants strains. Schematic presentation of the *hmu* and *hus* operons in the wild type W83 and Δ*hmuY*, Δ*husA*, Δ*hmuY*Δ*husA* mutant strains (A, B, D, E). Lack of HmuY production was confirmed by Western blotting using anti-HmuY antibodies (C). Deletion of the *husA* gene was confirmed by PCR using genomic DNA, separation of PCR product in 1% agarose gel, and staining with ethidium bromide (F). F1 and R, primers specific for the flanking regions of the *husA* gene, amplified DNA fragment in the wild type W83 strain comprising the *husA* gene with the flanking regions, and in the Δ*husA* mutant strain DNA fragment comprising additional sequence of the *tetQ* cassette. F2 and R primers were specific to amplify fragment containing *tetQ* region only. RT-PCR using primers for qPCR confirming lack of *husA* and *hmuY* transcript in mutant strains (G). Transcripts were examined by RT-PCR. PCR products were separated in 2% agarose gel and stained with ethidium bromide. Primers for *husB*, *hmuR* and *16S rRNA* served as controls. For RT-PCR analysis bacteria were grown in iron/heme rich conditions (Hm) for 24 hours. #1 and #2 indicate independent replicates.

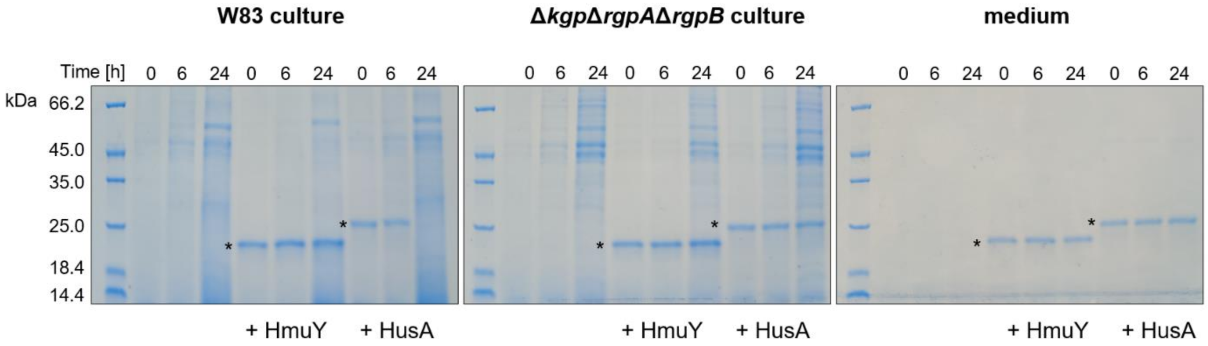


**FIG S2** Susceptibility of soluble forms of HmuY and HusA proteins to *P. gingivalis* proteases. Proteolytic digestion was examined by growing bacteria in high-iron/heme conditions (Hm) in the presence of 5 μM purified HmuY or HusA proteins (marked with asterisks). As a control, proteins were incubated with culture medium alone. Samples collected at the indicated time points were separated by SDS-PAGE and proteins were stained with CBB.

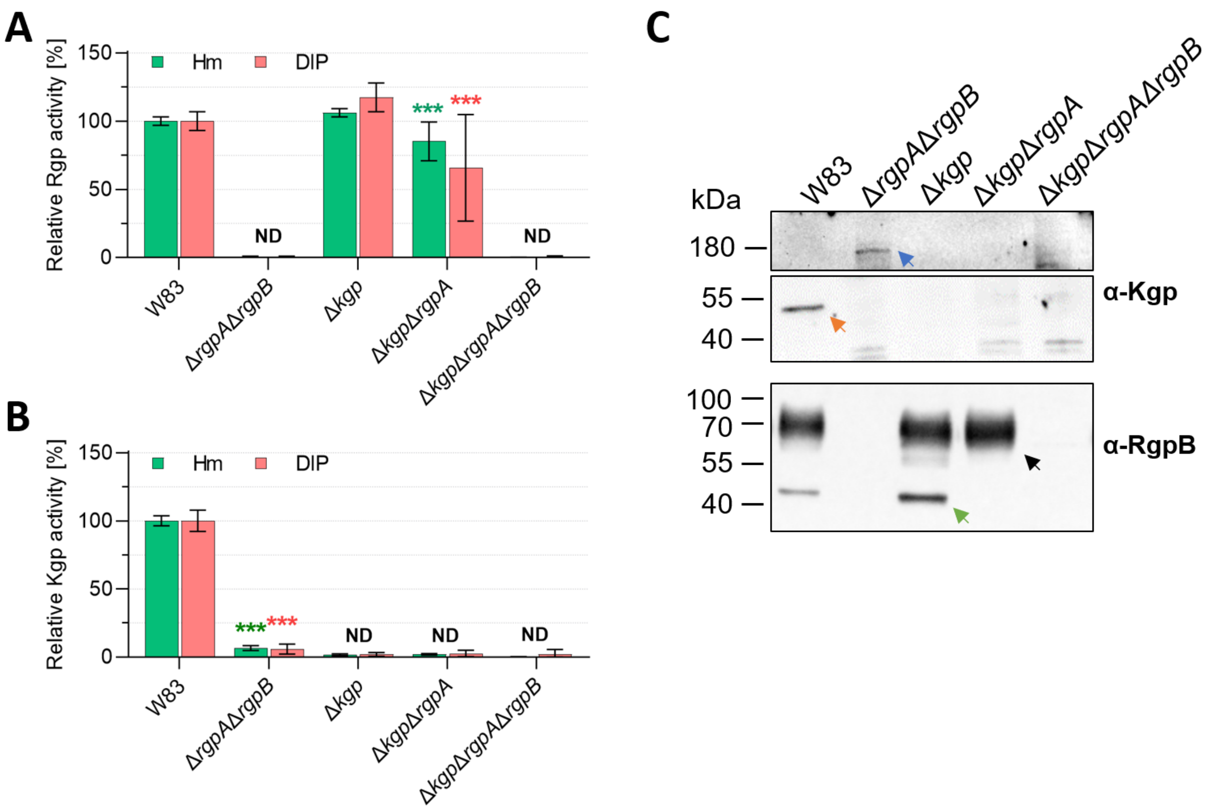


**FIG S3** Analysis of Rgp- and Kgp-specific activities in gingipain mutant strains. Proteolytic activity was determined in Δ*rgpA*Δ*rgpB*, Δ*kgp*, Δ*kgp*Δ*rgpA* and Δ*kgp*Δ*rgpA*Δ*rgpB* mutant strains in comparison to the wild type W83 strain. Bacteria were grown for 24 hours in iron/heme rich conditions (Hm) (A) or in iron/heme-depleted conditions (DIP) (B). Results for the wild type W83 strain served as a control set as 100%. ND – activity not detected. Experiments were carried out three times in two biological replicates and results are shown as mean values±standard deviation (mean±SD). ****P*<0.001. Presence of RgpB and Kgp, as well as processing of Kgp were verified by Western blotting using anti-Kgp (α-Kgp) or anti-RgpB (α-RgpB) antibodies (C). Gingipain forms are marked with arrows (blue - unprocessed Kgp form ~180 kDa, orange - Kgp catalytic domain ~ 48 kDa, black - unprocessed RgpB form ~70 kDa, green - Rgp catalytic domain ~45 kDa).

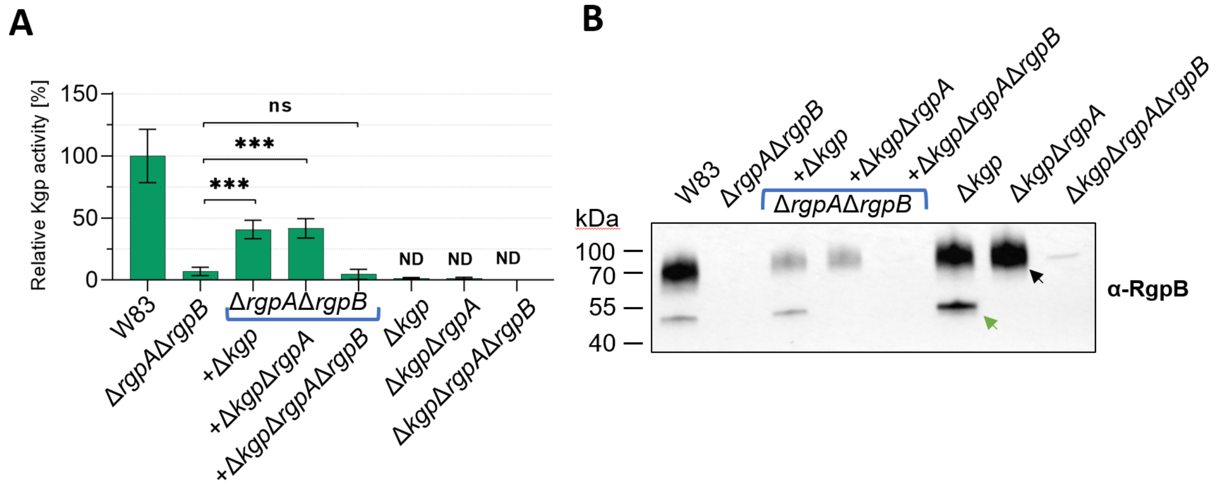


**FIG S4** Complementation of the lack of Rgp activity. Kgp-specific activity determined after 24 hours in Δ*rgpA*Δ*rgpB* cultures complemented with strains exhibiting Rgp activity but no Kgp activity (A). Results for the wild type W83 strain served as a control set as 100%. ND – activity not detected. Experiments were carried out three times. Results are shown as mean values±standard deviation (mean±SD); ns – statistically not relevant, ***$P$<0.001. Presence of RgpB by verified by Western blotting using anti-RgpB antibodies (α-RgpB) (B). Gingipain forms are marked with arrows (black - unprocessed RgpB form ~70 kDa, green - Rgp catalytic domain ~45 kDa).
